# Mutations in *rpoB* That Confer Rifampicin Resistance Can Alter Levels of Peptidoglycan Precursors and Affect β-Lactam Susceptibility

**DOI:** 10.1128/mbio.03168-22

**Published:** 2023-02-13

**Authors:** Yesha Patel, Vijay Soni, Kyu Y. Rhee, John D. Helmann

**Affiliations:** a Department of Microbiology, Cornell University, Ithaca, New York, USA; b Department of Medicine, Division of Infectious Diseases, Weill Cornell Medicine, New York, New York, USA; Pennsylvania State University

**Keywords:** *Bacillus subtilis*, *Mycobacterium tuberculosis*, RNA polymerases, antibiotic resistance, antibiotic synergy, β-lactams, metabolomics, peptidoglycan, rifampicin

## Abstract

Bacteria can adapt to stressful conditions through mutations affecting the RNA polymerase core subunits that lead to beneficial changes in transcription. In response to selection with rifampicin (RIF), mutations arise in the RIF resistance-determining region (RRDR) of *rpoB* that reduce antibiotic binding. These changes can also alter transcription and thereby have pleiotropic effects on bacterial fitness. Here, we studied the evolution of resistance in Bacillus subtilis to the synergistic combination of RIF and the β-lactam cefuroxime (CEF). Two independent evolution experiments led to the recovery of a single *rpoB* allele (S487L) that was able to confer resistance to RIF and CEF through a single mutation. Two other common RRDR mutations made the cells 32 times more sensitive to CEF (H482Y) or led to only modest CEF resistance (Q469R). The diverse effects of these three mutations on CEF resistance are correlated with differences in the expression of peptidoglycan (PG) synthesis genes and in the levels of two metabolites crucial in regulating PG synthesis, glucosamine-6-phosphate (GlcN-6-P) and UDP-*N*-acetylglucosamine (UDP-GlcNAc). We conclude that RRDR mutations can have widely varying effects on pathways important for cell wall biosynthesis, and this may restrict the spectrum of mutations that arise during combination therapy.

## INTRODUCTION

Bacteria adapt to environmental stresses by coordinated changes in transcription described as bacterial stress responses (1). However, when these phenotypic processes are overwhelmed, and most cells are either killed or growth inhibited, there is strong selective pressure for the emergence of adaptive mutations that confer resistance (2). Mutations in *rpoB*/*rpoC*, encoding the β and β′ subunits of the RNA polymerase (RNAP) core enzyme, can facilitate adaptation to a variety of environmental and antibiotic stresses (3–6). However, the pleiotropic nature of mutations affecting the core RNAP subunits has made it challenging to discern the specific basis of such phenotypes (7). One exception is rifampicin (RIF) resistance (8). RIF binds to the β-subunit of RNAP to suppress transcription, and substitutions in RpoB inhibit RIF binding, resulting in drug resistance (9). Importantly, such mutations are localized to the RIF-binding pocket and define a RIF resistance-determining region (RRDR). These RRDR changes dramatically reduce RIF binding and can also have other less well-understood effects on RNA polymerase function (10).

RRDR mutations affect the β-subunit of RNAP and often have collateral effects, such as reduced fitness (11) and altered susceptibility to other antibiotics (12). Accordingly, the ability of RIF to select RNAP mutations has been used as a tool for altering cell physiology (13). One possibility is that the mutant RNAP is altered in its biochemical properties or interactions with regulatory factors, and this leads to a change in the transcriptional landscape. For instance, selection of RIF resistance in Bacillus subtilis led to strains defective in sporulation, providing early support for the idea that the genetic program of sporulation might require modifications of RNAP (14). Further, altered expression of metabolic enzymes might account for the effects of *rpoB* mutations on the ability to grow on diverse carbon sources (15). In Mycobacterium tuberculosis (MTB), RIF-resistant *rpoB* mutants display an altered cell wall metabolism, perhaps due to effects on the channeling of metabolites into cell wall precursors (16, 17).

Since *rpoB* mutations may have global effects on cell physiology, the RRDR mutations that emerge in response to RIF selection can be influenced by other features of the growth environment. This phenomenon has been explored in B. subtilis, where both the frequency and spectrum of RRDR mutations are altered in diverse environments (including that of a spaceflight!) (18–20). In a clinical context, RIF is administered as part of a multidrug therapy for the treatment of MTB (21). Thus, it is important to consider the influence of other antibiotics on the acquisition of *rpoB* mutations conferring RIF resistance. More generally, it is important to understand the interactions between coadministered drugs and the impact of the evolution of resistance to one drug on susceptibility to the partner drug.

Here, we explore the physiological and genetic interactions between RIF and the cell wall-inhibiting β-lactam cefuroxime (CEF). Recently, β-lactams have been suggested as part of multidrug treatment regimens for drug-resistant tuberculosis (TB) (22). CEF is a potent β-lactam commonly used against B. subtilis (23), a model organism used in the current study. We demonstrated that RIF and CEF are synergistic against B. subtilis, and coselection with these two antibiotics led only to RRDR mutations. However, under these coselection conditions, the spectrum of RRDR mutations was reduced. When commonly arising RRDR mutations were characterized, only one mutation was identified to simultaneously confer high-level RIF and CEF resistance. The effects of RRDR mutations on CEF resistance correlated with changes in the expression of peptidoglycan synthesis enzymes and the levels of key intermediates. These findings highlight the ability of RRDR mutations to have divergent effects on microbial physiology.

## RESULTS

### Rifampicin (RIF) and cefuroxime (CEF) exhibit synergy against B. subtilis.

**FIG 1:**
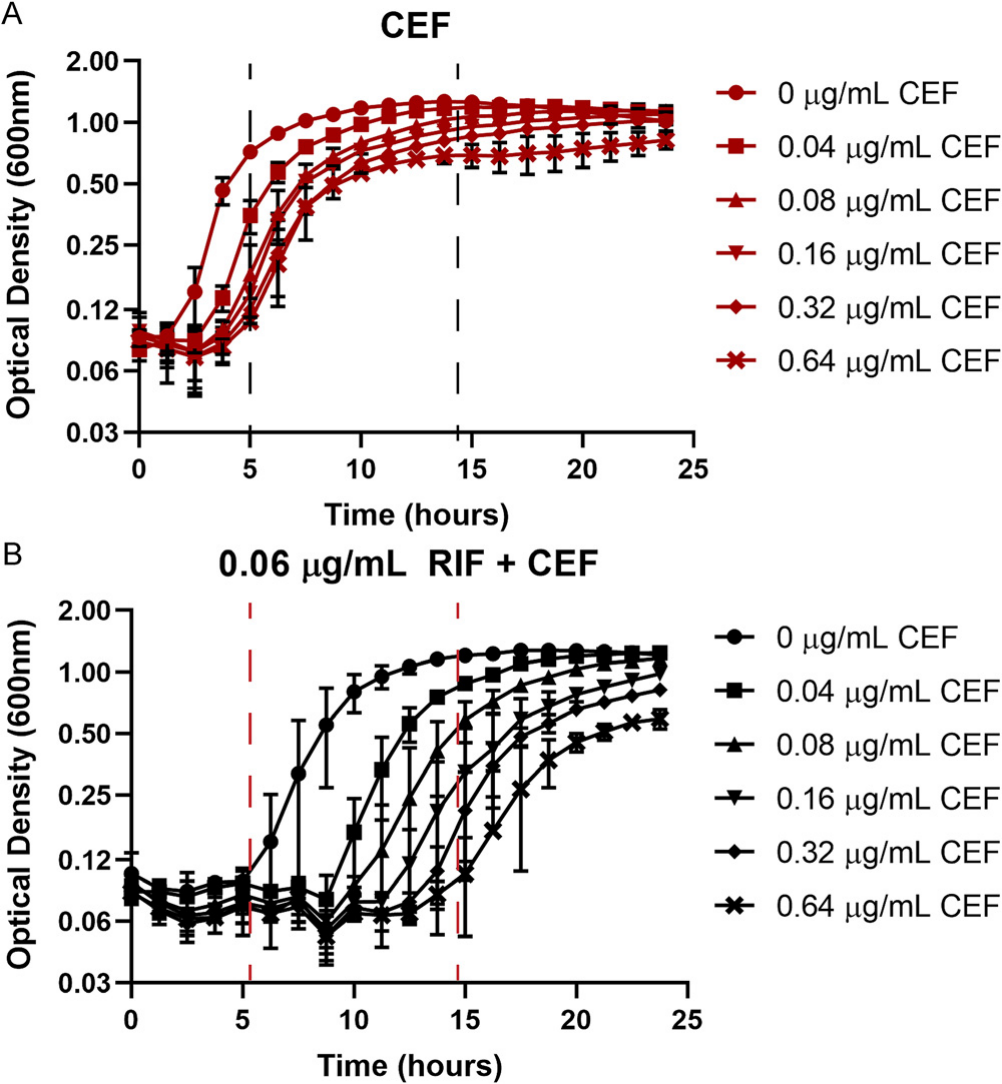
(A and B) Synergy between rifampicin (RIF) and cefuroxime (CEF) monitored by growth kinetics. Cell density was monitored after treatment with sub-MIC levels of CEF alone (A) or in the presence of 0.06 μg/mL RIF (B). The observed lag phases were all less than 5 h with CEF alone and increased to nearly 15 h with the combination treatment, as highlighted by the dashed lines.

A synergistic interaction between β-lactams and RIF has been reported against Gram-positive bacteria, including both methicillin-resistant staphylococci (24) and mycobacteria (25). We chose to test for synergy in B. subtilis between RIF and the cephalosporin cefuroxime (CEF). CEF, with an MIC of 5.12 μg/mL (Fig. S1A in the supplemental material), acts by preferentially binding to and inhibiting the activity of class A penicillin-binding proteins (PBPs), enzymes involved in the polymerization of peptidoglycan (PG) precursors (26). Using a checkerboard assay, we found the combination of RIF and CEF to be strongly synergistic with a zero-interaction potency (ZIP) score (27) of >10 over a range of antibiotic concentrations (Table 1; Table S3). Values for the combination of 0.06 μg/mL RIF with increasing concentrations of CEF have been listed in the table for illustration. The full data set, including other concentrations, is available in Table S3. On treatment with sub-MICs of CEF (up to 0.64 μg/mL), the lag phase was increased by no more than 3 h (Fig. 1A). A sub-MIC of RIF (0.06 μg/mL; Fig. S1B) also led to an increase in lag phase (from <1.5 h to ~5 h). However, these cells were now very sensitive to growth inhibition by CEF, with as little as 0.08 μg/mL CEF leading to a lag phase of ~10 h (Fig. 1B). Similarly, the presence of sub-MIC CEF (0.64 μg/mL) reduced the RIF MIC by 4-fold from 0.125 to 0.03 μg/mL (Fig. S1B and C). This change corresponds to a fractional inhibitory concentration index (FICI) (28) of 0.36, further supporting the conclusion that these two antibiotics act synergistically.

**TABLE 1:** ZIP scores for the combination of 0.06 μg/mL RIF with increasing concentrations of CEF

RIF (μg/mL)	CEF (μg/mL)	ZIP score
0.06	0	0.0
0.06	0.04	61.7
0.06	0.08	69.1
0.06	0.16	65.7
0.06	0.32	55.8
0.06	0.64	43.1
0.06	1.28	27.6
0.06	2.56	14.3
0.06	5.12	5.2
0.06	10.24	0.0

10.1128/mbio.03168-22.3 TABLE S3 Synergy determination between RIF and CEF by ZIP score. Download Table S3, DOCX file, 0.02 MB. Copyright © 2023 Patel et al. 2023 Patel et al. https://creativecommons.org/licenses/by/4.0/ This content is distributed under the terms of the Creative Commons Attribution 4.0 International license.

10.1128/mbio.03168-22.4 FIG S1 MICs determined by growth kinetics. (A to C) Cell density was monitored after treatment with CEF (A), RIF (B), or a combination of 0.64 μg/mL CEF with RIF at concentrations mentioned in the legend (C). MIC is defined as >90% growth inhibition compared to untreated control after 8 h of treatment. Download FIG S1, TIF file, 2.4 MB. Copyright © 2023 Patel et al. 2023 Patel et al. https://creativecommons.org/licenses/by/4.0/ This content is distributed under the terms of the Creative Commons Attribution 4.0 International license.

### Cotreatment with RIF and CEF selects for mutations in *rpoB*.

**FIG 2:**
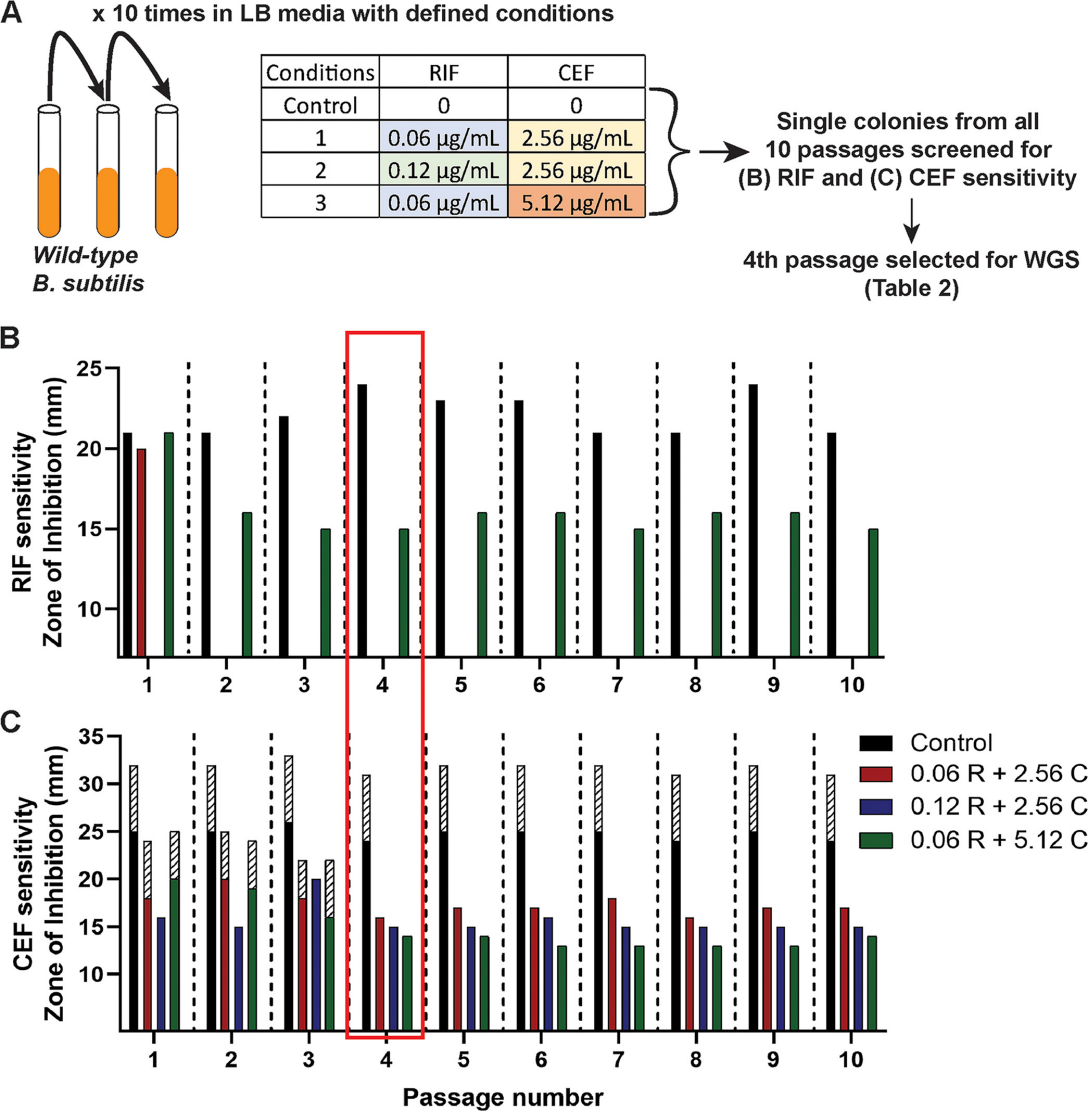
Evolution of WT B. subtilis to achieve RIF and CEF resistance. (A) Schematic of the evolution experiment performed in the presence of the RIF + CEF combination; WGS, whole-genome sequencing. (B and C) RIF (B) and CEF (C) susceptibilities as measured by zone of inhibition for the 10 passages evolved under the 3 combination treatments. The three RIF and CEF concentrations used for evolving the cells are mentioned in the legend. The control group consists of 10 passages of WT cells that have not been treated with any drugs. The shaded bars in C represent the zone of lower density.

Drug synergy is a clinically attractive feature of antibiotic chemotherapy. However, drug interactions also have the potential to influence the evolution of resistance (29). Both RIF and CEF susceptibility is influenced by mutations in RNA polymerase (30, 31). We therefore sought to explore how cotreatment with both RIF and CEF affected the evolution of resistance. We hypothesized that the combination of RIF and CEF might select for the emergence of mutations at novel loci. We evolved B. subtilis by repeated passage (10 times) in the presence of three alternative drug combinations (Fig. 2A): 0.06 μg/mL RIF with 2.56 μg/mL CEF (0.5× MIC of the individual drugs), 0.12 μg/mL RIF with 2.56 μg/mL CEF (MIC of RIF and 0.5× MIC of CEF), and 0.06 μg/mL RIF with 5.12 μg/mL CEF (0.5× MIC of RIF and MIC of CEF). Under all three conditions, cells developed resistance to both drugs by the fourth passage, as measured by a decrease in diameter in a zone of inhibition (ZOI) assay (Fig. 2B and C). The absence of red and blue bars in Fig. 2B represents complete loss of the ZOI and hence high resistance to RIF. Interestingly, when evolved in the presence of the highest CEF concentration (5.12 μg/mL), cells were only able to acquire low-level RIF resistance.

To identify the genetic changes associated with resistance, we performed whole-genome sequencing (WGS) of single colonies recovered from the fourth passage of selection. Interestingly, all three evolved strains had mutations in the RRDR of *rpoB* (Table 2). This suggests that even in the presence of two drugs, the most facile path to resistance to both drugs is through alterations in the RRDR region of *rpoB.* The two independently evolved strains (A and B) that were selected with sub-MIC levels of CEF both acquired high-level RIF resistance with an identical mutation, S487L. The RRDR region is highly conserved (32), and this mutation corresponds to S531L in Escherichia coli and S450L, which is the most commonly occurring RIF resistance mutation in M. tuberculosis (33). Strain C, evolved with CEF at its MIC (5.12 μg/mL), acquired an *rpoB* P520L mutation that contributed comparatively low-level RIF resistance (34). This suggests that the selective pressure imposed by higher CEF concentrations might preclude the acquisition of high RIF resistance through typical RRDR mutations. We sought to confirm this finding by repeating the experiment with 10 additional biological replicates. Five tubes were grown with 0.06 μg/mL RIF and 5.12 μg/mL CEF (1× MIC) and five tubes with 0.06 μg/mL RIF and 10.24 μg/mL CEF (2× MIC). In support of the previous experiment, none of the strains acquired high RIF resistance even after 10 passages. Sequencing of the RRDR region from eight isolates led to four strains with atypical RRDR region mutations that led to modest increases in RIF and high CEF resistance (L489S, A478V [2 isolates], and S468P) and four that did not contain RRDR mutations. Thus, high levels of CEF seem to impede the emergence of most RRDR region mutations that are known to confer high-level RIF resistance in favor of mutations that confer CEF resistance and only partial RIF resistance.

**TABLE 2:** The mutations identified by whole-genome sequencing after evolution

Drug combination	Gene	Coding region change	Amino acid change
Strain A (0.06 R + 2.56 C)	*rpoB*	1460 C > T	S487L
Strain B (0.12 R + 2.56 C)	*rpoB*	1460 C > T	S487L
Strain C (0.06 R + 5.12 C)	*rpoB*	1559 C > T	P520L

### *rpoB* mutants exhibit altered susceptibility to other cell wall-acting antibiotics.

**FIG 3:**
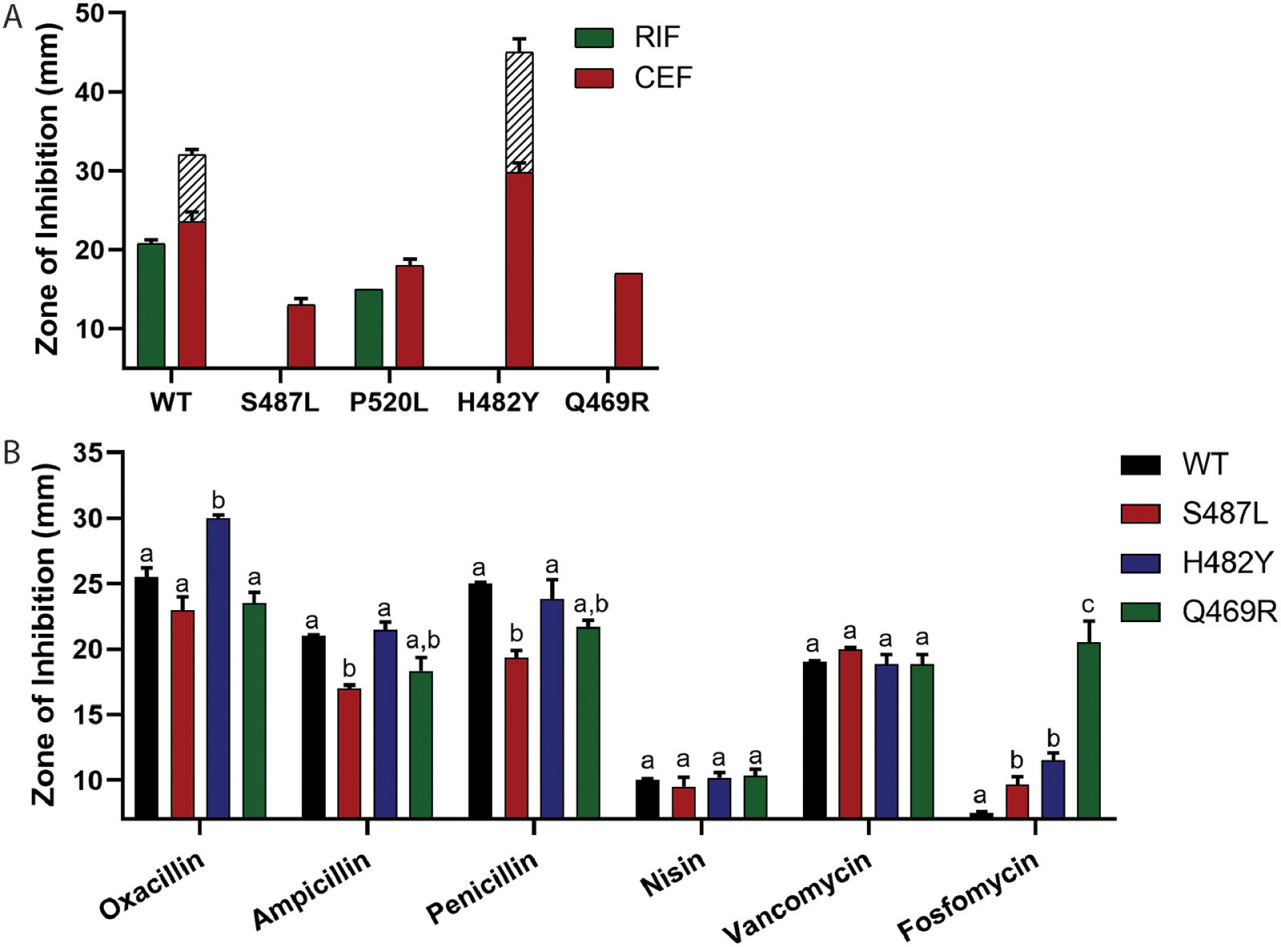
Drug susceptibilities of *rpoB* mutants. (A) Zone of inhibition against RIF and CEF for different *rpoB* mutants (note that only P520L had a detectable inhibition zone with RIF). (B) Zone of inhibition for β-lactams oxacillin, ampicillin, and penicillin and other cell wall-inhibiting drugs, such as nisin, vancomycin, and fosfomycin for the common clinically associated RIF-resistant *rpoB* mutants. Significance was defined as a *P* value of <0.001. A comparison was done between all mutants treated with the same drug. No comparison was done between drugs. A strain with a significant difference compared to others is given a different letter, and “a,b” indicates a value that is not significantly different from those strains in either group a or group b.

In addition to characterizing RIF-resistant mutants selected by both RIF and CEF (Table 2), we also isolated *rpoB* mutants on agar containing high concentrations (512 μg/mL) of RIF alone. Two additional mutations (H482Y and Q469R) were recovered, which have been identified in prior studies of RIF resistance in B. subtilis (35). Mutations in the RRDR residues corresponding to B. subtilis S487, H482, and Q469 (Table 3) correspond to more than 90% of RIF-resistant MTB clinical isolates (36). Because of the clinical prevalence of these mutations and the cross-resistance of the S487L mutant to CEF, we characterized the CEF sensitivity of the H482Y and Q469R RIF-resistant mutants (Table 3; Fig. 3A). In contrast to mutants evolved under combination selection (S487L), the H482Y mutation made cells highly susceptible to CEF (32 times more sensitive than wild type [WT]), whereas the Q469R mutation led to a modest increase in CEF resistance (2 times more resistant than WT). Combination treatment using RIF and β-lactams has been proposed as a potential drug therapy for M. tuberculosis (37). We therefore tested whether two common RIF-resistant mutations in M. tuberculosis (S450L and H445Y) also alter CEF susceptibility. Indeed, both S450L and H445Y were 2- to 4-fold more sensitive to CEF than to H37Rv.

**TABLE 3:** RIF and CEF MICs of different *rpoB* mutants

Mutation (B. subtilis)	E. coli locus	M. tuberculosis locus	RIF MIC^*a*^ (μg/mL)	CEF MIC^*a*^ (μg/mL)
WT			0.125	5.12
H482Y	H526Y	H445Y	**>4**	*0.16*
Q469R	Q513R	Q432R	**>4**	10.24
S487L	S531L	S450L	**>4**	**20.48**
P520L	P562L	P481L	4	**20.48**

a Values are shaded in gray to define resistance. Dark gray with bold represents higher resistance. Values in italic font represents increased susceptibility for the drug.

Although H482Y frequently emerges in cells subject to RIF selection, this mutation is disfavored in the presence of CEF because it greatly increases CEF sensitivity (Fig. 3A). Such interactions, where emergence of resistance to one antibiotic increases the susceptibility to another, are beneficial in combination therapies (38). We next tested the sensitivity of the three clinically relevant RIF resistant mutants toward additional β-lactams and other antibiotics that target the cell wall (Fig. 3B). All β-lactams inhibit the formation of the PG layer by targeting different PBPs with different affinities (23). Three additional β-lactams (oxacillin, ampicillin, and penicillin) were similar to CEF, with S487L and Q469R increasing resistance and H482Y conferring sensitivity. Neither effect was as strong as for CEF, which can be attributed to CEF having the highest affinity for PBP1, the most abundant and primary class A PBP (26).

Extending beyond β-lactams, we also tested the sensitivity of the mutants for nisin and vancomycin, both of which bind lipid II and prevent PG synthesis and cross-linking and, in the case of nisin, can form membrane pores (39, 40). Compared to WT, none of the *rpoB* mutants had a significant difference in sensitivity toward either of these drugs (Fig. 3B). In contrast, all the mutants (and especially Q469R) were more susceptible toward fosfomycin (Fig. 3B), which inhibits the MurA-dependent synthesis of UDP-*N*-acetylmuramic acid from UDP-*N*-acetylglucosamine (UDP-GlcNAc) (41). As a control, we also tested the sensitivity of the mutants against drugs acting on other cellular processes, including chloramphenicol, which inhibits protein synthesis (42), triclosan, which inhibits fatty acid synthesis (43), and paraquat, which generates reactive oxygen species (ROS) toxicity in the cells (44). None of the mutants had a significant difference in the sensitivity against these drugs (Fig. S2). In conclusion, the predominant *rpoB* mutations associated with high RIF resistance had various levels of sensitivity to drugs that inhibit PG synthesis.

10.1128/mbio.03168-22.5 FIG S2 Drug susceptibilities of *rpoB* mutants as measured by zone of inhibition against drugs that do not directly target cell wall synthesis. Chloramphenicol inhibits protein synthesis, triclosan inhibits fatty acid synthesis, and paraquat generates ROS toxicity in the cells. Download FIG S2, TIF file, 0.2 MB. Copyright © 2023 Patel et al. 2023 Patel et al. https://creativecommons.org/licenses/by/4.0/ This content is distributed under the terms of the Creative Commons Attribution 4.0 International license.

### *rpoB* mutations alter the expression of genes affecting PG synthesis.

**FIG 4:**
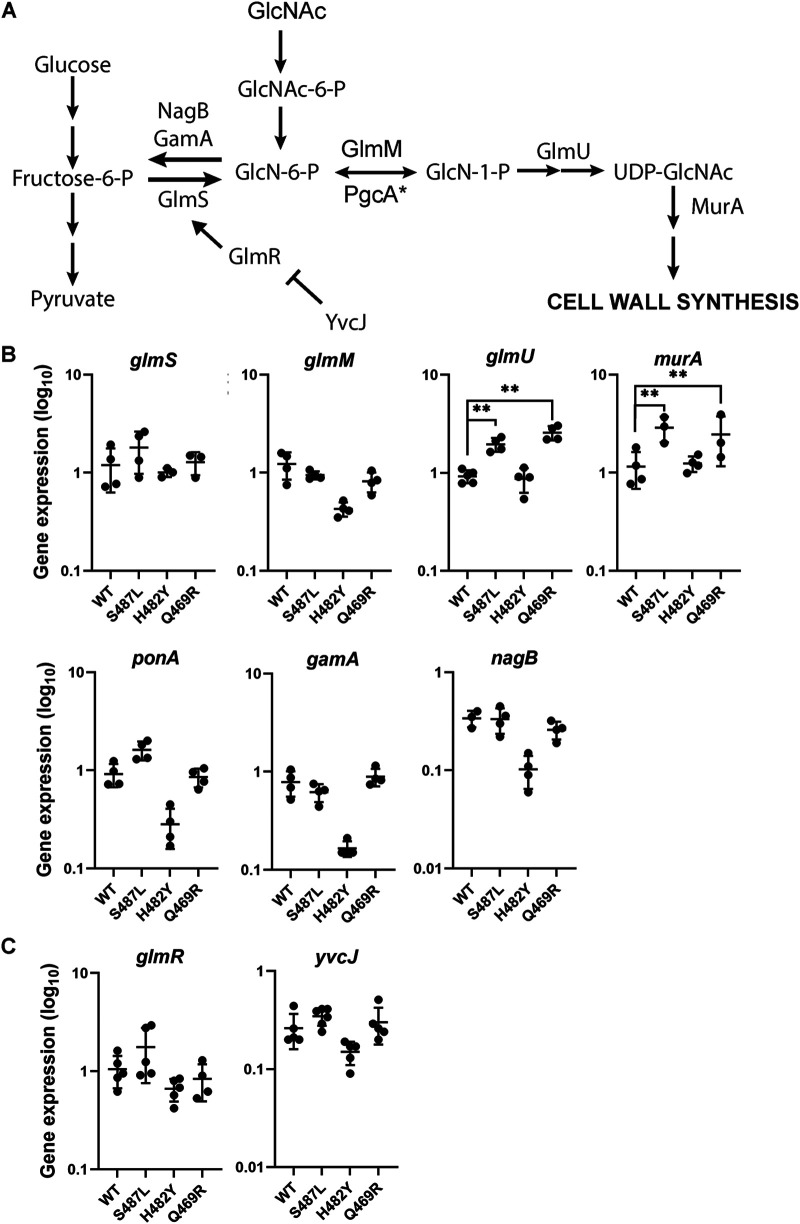
The effect of *rpoB* mutations on peptidoglycan (PG) synthesis. (A) Schematic of the PG synthesis pathway. (B and C) Expression levels of enzymes (B) and regulators (C) involved in PG synthesis in WT and *rpoB* mutants S487L, H482Y, and Q469R as determined by real-time PCR. The expression levels were calculated by the 2^−Δ^*^CT^* method. *gyrA* was used as the internal control to normalize the levels of the genes of interest. The values are plotted on a log_10_ scale. Significance was calculated by two-way ANOVA with Tukey’s multiple-comparison test. The two asterisks (**) indicate *P* values less than 0.001.

Based on our antibiotic sensitivity results, we hypothesized that these RRDR mutations may change the interaction of RNAP with promoters or regulators involved in the expression of PG synthesis genes. We therefore sought to evaluate the transcript levels of representative PG synthesis genes (*glmS*, *glmM*, *glmU*, *murA*, and *ponA*) and two genes that function to divert PG intermediates back into glycolysis (*gamA* and *nagB*) (Fig. 4A). PG synthesis branches from the fructose-6-phosphate (fructose-6-P) node in glycolysis when GlmS converts fructose-6-phosphate to glucosamine-6-phosphate (GlcN-6-P) (45, 46). GlcN-6-P is isomerized by GlmM into GlcN-1-P, which is converted by GlmU to UDP-GlcNAc. MurA initiates synthesis of the second sugar required for PG synthesis, UDP-MurNAc. We included *ponA*, which encodes PBP1, the primary class A PBP involved in PG synthesis during vegetative growth and a major target of CEF inhibition (23, 26). PG synthesis can also be supported by import of amino sugars such as GlcNAc present in the growth medium. Catabolism of GlcNAc leads to GlcN-6-P, a branchpoint metabolite that can be used by GlmM to support PG synthesis or, when in excess, can be routed into glycolysis through the GamA (47) and NagB (48) enzymes (Fig. 4A).

In the case of the CEF-resistant (CEF^R^) S487L and Q469R mutants, *glmU* and *murA* were expressed at significantly higher levels than in WT cells, and other tested genes were unchanged (Fig. 4B). CEF resistance was notably not correlated with upregulation of *ponA*, encoding a major target for CEF. In the case of the CEF-sensitive (CEF^S^) H482Y mutant, *glmM* and *ponA* were expressed at lower levels than in WT cells. We hypothesized that these reduced *glmM* levels might be correlated with an increase in expression of *gamA* and *nagB*. However, in the H482Y mutant mRNA levels of the latter genes were reduced relative to those observed in WT. None of the mutants had a difference in their growth kinetics in the absence of any drug (Fig. S3), suggesting that the altered drug sensitivity of the mutants did not result from slower growth. Thus, we conclude that CEF resistance is correlated with increased transcript levels for some enzymes in PG synthesis (*glmU* and *murA*), whereas sensitivity is correlated with reduced mRNA levels for other enzymes (*glmM*, *ponA*, *gamA*, and *nagB*). Whether these changes in mRNA levels are due to effects of RRDR mutations on RNAP activity at the corresponding promoters or are an indirect effect of other changes in metabolism is not yet clear.

10.1128/mbio.03168-22.6 FIG S3 Growth kinetics of *rpoB* mutants in LB medium in the absence of any drug treatment. Download FIG S3, TIF file, 0.2 MB. Copyright © 2023 Patel et al. 2023 Patel et al. https://creativecommons.org/licenses/by/4.0/ This content is distributed under the terms of the Creative Commons Attribution 4.0 International license.

Metabolic flux can be regulated by changes in enzyme activity or enzyme expression. For PG synthesis, GlmS is under complex regulation. The level of *glmS* mRNA is regulated by GlcN-6-P-activated mRNA cleavage by the *glmS* ribozyme (49). However, levels of *glmR* mRNA were only modestly different between RRDR mutants and those observed in WT (Fig. 4C). In addition, GlmS activity is allosterically activated by GlmR (50). GlmR activity is antagonized by complex formation with YvcJ in the presence of high UDP-GlcNAc (51). Therefore, we sought to determine whether RRDR mutations affect the levels of metabolites that might impact PG synthesis.

### RRDR mutations alter the levels of key PG intermediates.

**FIG 5:**
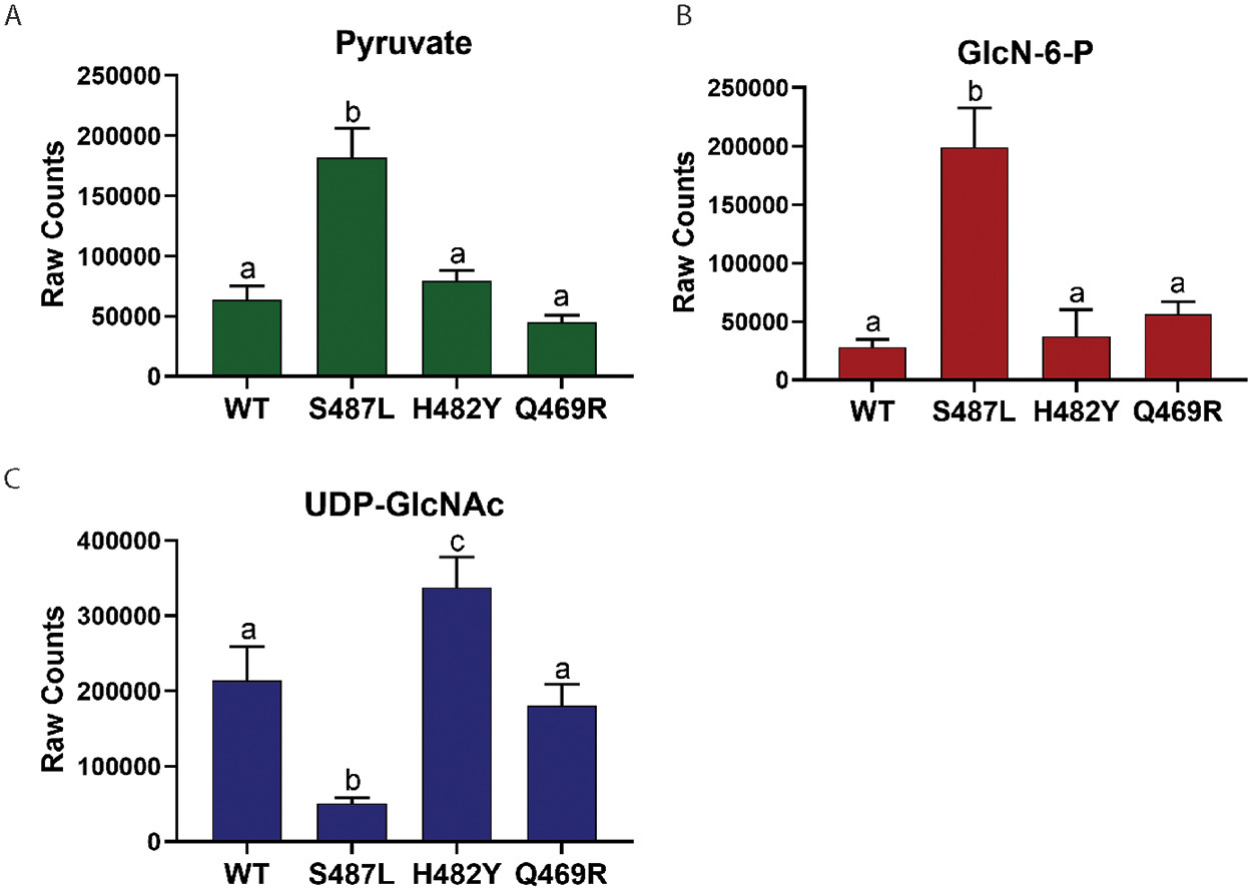
Metabolite levels in *rpoB* mutants. (A) Metabolite levels of pyruvate, which indicate the flux through glycolysis. (B) Metabolite levels of GlcN-6-P, which determine flux into PG synthesis. (C) Metabolite levels of UDP-GlcNAc, which indicate the rate of PG formation. Same letters define a data set with no significant difference. Significance was defined as a *P* value of <0.0001. A comparison was performed between all strains for each metabolite. A strain with a significant difference compared to others is given a different letter.

To monitor the impact of RRDR mutations on metabolite pools, we performed untargeted metabolomics. We focused our attention on examining the levels of the two key regulatory intermediates noted above, GlcN-6-P and UDP-GlcNAc (Fig. 4A), and pyruvate, which is indicative of the flux of fructose-6-phosphate into glycolysis (52). The Q469R strain did not show any significant difference in the levels of these metabolites, so we focused on the differences between the CEF^R^ (S487L) and CEF^S^ (H482Y) strains (Fig. 5).

For the CEF^R^ S487L mutant, we observed an increase in GlcN-6-P and a decrease in UDP-GlcNAc. Since UDP-GlcNAc regulates GlmS activity through the YvcJ/GlmR pathway (Fig. 4A), low UDP-GlcNAc will lead to high GlmS activity, which might account for elevated GlcN-6-P. We also noted elevated mRNA levels for *glmU* and *murA* (Fig. 4B). Thus, we conclude that the S487L mutant has changes in both gene expression and metabolite levels, consistent with a higher rate of PG synthesis. Although one might expect that elevated GlcN-6-P could reduce *glmS* mRNA levels (by ribozyme cleavage) and increase the expression of *gamA* and *nagB*, our real-time PCR results showed no evidence for these changes (Fig. 4B), suggesting that GlcN-6-P has not reached levels needed to trigger these responses.

In contrast, the CEF^S^ H482Y mutant had elevated levels of UDP-GlcNAc. In this case, we predict that the high UDP-GlcNAc will cause sequestration of GlmR in a YvcJ:GlmR:UDP-GlcNAc complex and thereby prevent GlmR stimulation of GlmS activity (51). By restricting GlmS activity, this could reduce flux of fructose-6-P into PG and contribute to the CEF-sensitive phenotype. Thus, the most striking correlation to emerge from the metabolomics analysis is the correlation between UDP-GlcNAc and CEF sensitivity. Further, our data support the idea that a key function of UDP-GlcNAc is as a feedback regulator of GlmS activity, as mediated by the GlmR/YvcJ pathway (51).

### The ability of UDP-GlcNAc to modulate PG synthesis is dependent on GlmR.

**FIG 6:**
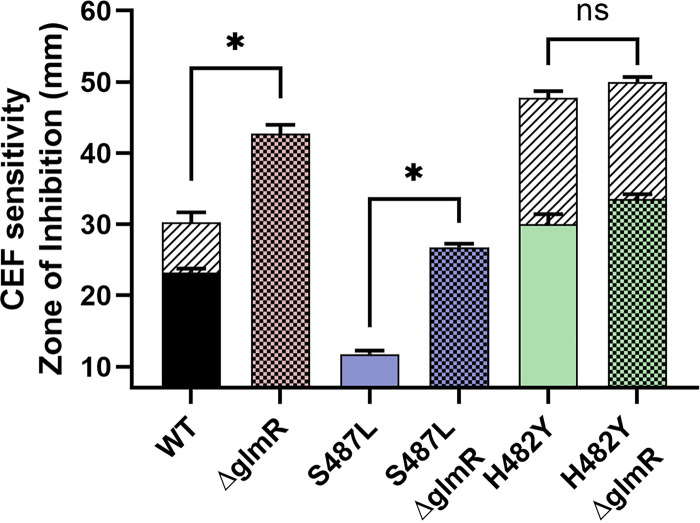
The importance of GlmR activity in CEF sensitivity. The sensitivity of WT and *rpoB* mutants with and without the deletion of *glmR* as measured by zone of inhibition. An asterisk (*) indicates *P* values less than 0.0001; ns, not significant.

We used epistasis studies to determine if the correlation of UDP-GlcNAc levels and CEF sensitivity is in fact mediated by the role of UDP-GlcNAc as a negative regulator of GlmR activity. The CEF^R^ S487L mutant has reduced UDP-GlcNAc levels that could result in increased activity of the GlmR regulator, and this, in turn, could lead to elevated PG synthesis and contribute to antibiotic resistance. Consistent with this model, the elevated CEF^R^ of the S487L mutant is lost in a strain additionally lacking *glmR* (Fig. 6). Conversely, in the CEF^S^ H482Y mutant, UDP-GlcNAc levels are high, and, therefore, we predict that GlmR will be largely nonfunctional due to sequestration in a YvcJ:GlmR:UDP-GlcNAc complex (51). Both the H482Y and the *glmR* mutations individually make cells CEF^S,^ but these two mutations are not additive in the H482Y *glmR* double mutant (Fig. 6.). This supports our hypothesis that H482Y and *glmR* function in the same pathway and that H482Y has effectively inactivated GlmR function by altering metabolism, leading to a high level of UDP-GlcNAc.

### Perturbing flux of amino sugars can alter CEF sensitivity.

**FIG 7:**
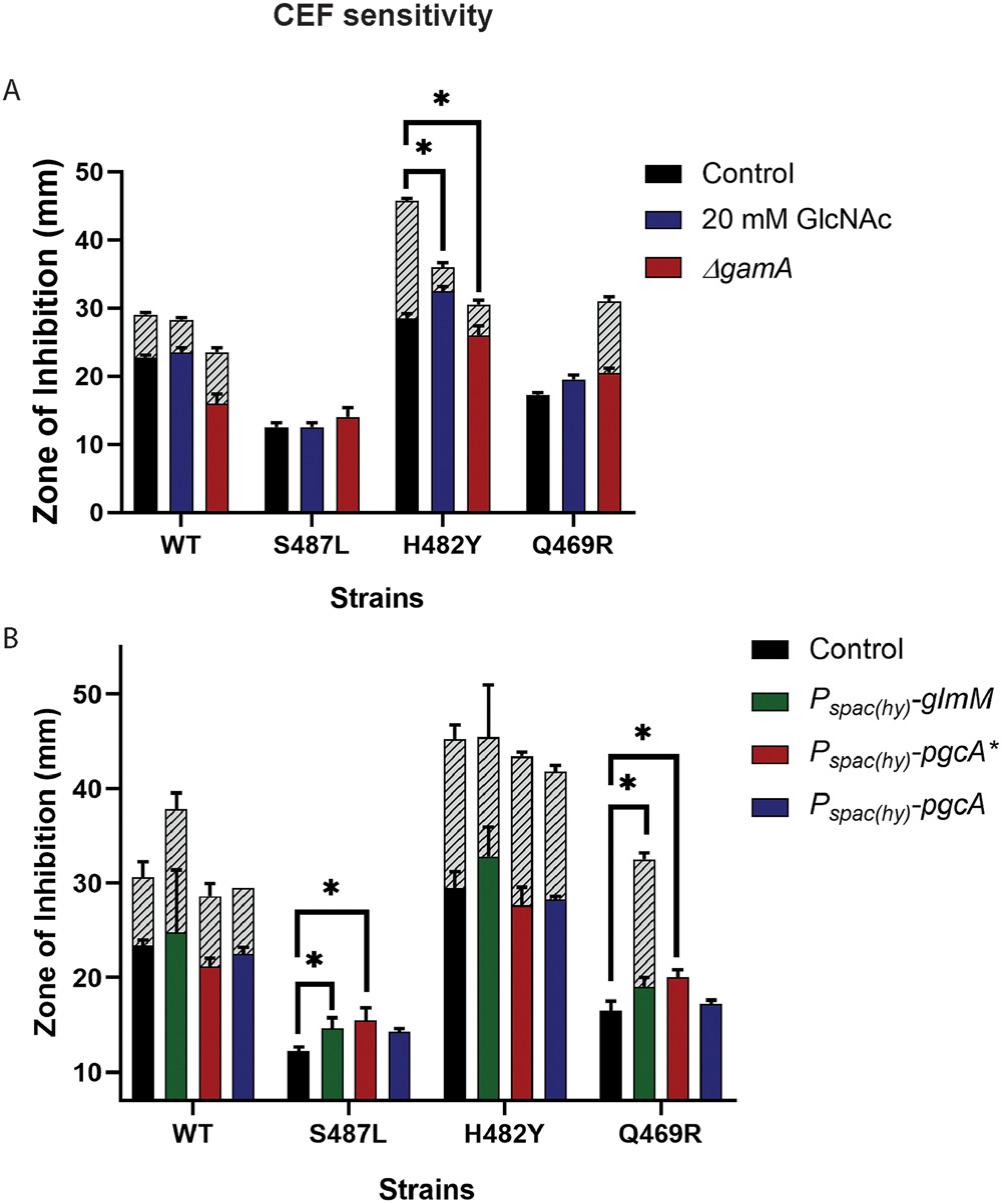
Perturbation of GlcN-6-P and UDP-GlcNAc levels in cells. (A and B) The sensitivity of WT and *rpoB* mutants against CEF as measured by zone of inhibition on medium supplemented with 20 mM GlcNAc and on deletion of *gamA*, which directs GlcN-6-P toward glycolysis (A), and after induction of the phosphoglucosamine mutase *glmM* and pgcA* and phosphoglucomutase *pgcA* (B). An asterisk (*) indicates *P* values less than 0.01.

We hypothesize that the CEF sensitivity of the H482Y mutant is due to restricted GlmS activity resulting from elevated UDP-GlcNAc levels. Therefore, we sought to bypass GlmS by supplementing cells with GlcNAc, which has been shown to increase the level of GlcN-6-P (53). Indeed, in the presence of GlcNAc, there was a significant increase in CEF resistance for the H482Y mutant (Fig. 7A). The growth of the cells in liquid medium in the presence of 0.04 μg/mL CEF was also significantly better when LB was supplemented with GlcNAc (Fig. S4). These results suggest that increasing flux of sugars into PG synthesis restores CEF resistance to H482Y by bypassing GlmS. Consistently, if we instead delete *gamA* (Fig. 4A), the flux of amino sugars present in the growth medium into glycolysis is restricted, and this also increases CEF resistance. We next tested the impact of increasing the flow of GlcNAc into UDP-GlcNAc on CEF resistance. We ectopically induced expression of the GlmM phosphoglucosamine mutase (PNGM) and PgcA*, an allele of phosphoglucomutase with increased PNGM activity (54). Neither gene was able to increase CEF resistance (Fig. 7B). This is consistent with the hypothesis that GlmS activity is restricted, GlcN-6-P is a limiting metabolite for PG synthesis, and only the import of amino sugars from outside the cell can bypass this restriction.

10.1128/mbio.03168-22.7 FIG S4 Growth kinetics of WT and the H482Y mutant. Growth was monitored in LB (control), LB with 0.04 μg/mL CEF, LB supplemented with GlcNAc, and LB supplemented with GlcNAc and 0.04 μg/mL of CEF. Download FIG S4, TIF file, 0.3 MB. Copyright © 2023 Patel et al. 2023 Patel et al. https://creativecommons.org/licenses/by/4.0/ This content is distributed under the terms of the Creative Commons Attribution 4.0 International license.

Conversely, the CEF^R^ S487L mutant did not exhibit any difference in CEF sensitivity in the presence or absence of 20 mM GlcNAc or after deletion of *gamA* (Fig. 7A). This is consistent with our hypothesis that this strain is not restricted in the flux of fructose-6-P into GlcN-6-P. In this case, induction of *glmM* or pgcA* actually led to a slight increase in CEF sensitivity. In contrast, induction of PgcA, which has comparatively low PNGM activity (54), had no effect (Fig. S5). We speculate that with this strain, which has high GlcN-6-P levels (Fig. 7B), further increases in the synthesis of amino sugars leads to a metabolic imbalance. Finally, for the Q469R mutant, which did not exhibit any significant depletion or accumulation of the PG intermediates, GlcNAc addition did not change CEF susceptibility. Similar to S487L, induction of *glmM* or pgcA* in Q469R also led to a slight increase in CEF sensitivity (Fig. 7B).

10.1128/mbio.03168-22.8 FIG S5 CEF susceptibility as measured by zone of inhibition assay for WT and *rpoB* mutants. Black control bars represent the CEF sensitivity of the mutants. Blue *pgcA* bars represent the CEF sensitivity of the respective strains with the *pgcA* gene deleted. Download FIG S5, TIF file, 0.1 MB. Copyright © 2023 Patel et al. 2023 Patel et al. https://creativecommons.org/licenses/by/4.0/ This content is distributed under the terms of the Creative Commons Attribution 4.0 International license.

## DISCUSSION

Drug interactions have a strong impact on the evolution of resistance (55). Here, we evaluated the emergence of resistance to a combination of a β-lactam (CEF) and rifampicin (RIF). These two drugs are synergistic in B. subtilis, as shown also for other bacteria (56–59). We used *in vitro* evolution followed by whole-genome sequencing to identify mutations that enable growth in the presence of this dual selection. Strikingly, only one single RRDR mutation (S487L) emerged that confers high-level resistance to both antibiotics. With CEF at or above the MIC, the acquisition of high-level RIF resistance was restricted. When this selection was repeated and colonies were screened specifically for RRDR mutations, we identified several other mutations not commonly associated with RIF resistance that confer high-level CEF resistance and only modestly increase RIF resistance.

These results highlight the importance of RRDR mutations in RIF resistance (by reducing RIF binding to the β-subunit) and the ability of *rpoB* mutations to also confer resistance to other antibiotics by less direct mechanisms. In the presence of CEF, only a limited set of mutations can simultaneously lead to CEF and RIF resistance, and these were found in the RRDR. In fact, other common RRDR mutations that confer high-level RIF resistance were either sensitive (H482Y) or had lower resistance to CEF (Q469R). In MTB, both mutants corresponding to S487L and H482Y were sensitive to CEF compared to WT. The collateral sensitivity to CEF on acquiring RIF resistance is favorable when considering multidrug treatment (60). Further, cotreatment with β-lactams and RIF may constrain emergence of RIF resistance.

Mutations in *rpoB* that emerge in response to antibiotic selection can have broad effects on cell physiology (10, 61). Selection with RIF leads to RRDR mutations that often result in a significant decrease in cell fitness (62, 63), which leads to the emergence of compensatory mutations (64). Similarly, *rpoB* mutations have been described that alter susceptibility to cell wall-inhibiting drugs, such as β-lactams (6, 30), vancomycin, and daptomycin (65), although these mutations typically do not map to the RRDR (6). However, some RIF-resistance mutations in the RRDR not only decrease RIF binding but also lead to alterations in the cell wall (16). In E. coli, the clinically relevant H526Y RRDR mutant is very sensitive to cell wall inhibitors and to the deletion of genes encoding auxiliary functions related to cell wall synthesis and division (66). Similarly, we report here that B. subtilis RRDR mutations can lead to either sensitivity or resistance to an antibiotic (CEF) that inhibits PG synthesis.

The identification of S487L (CEF^R^) and H482Y (CEF^S^) mutants in B. subtilis presents a useful tool to understand the impact of RRDR mutations on cell wall homeostasis. Using transcriptomic and metabolomic studies, we present evidence for the importance of altered metabolite levels (GlcN-6-P and UDP-GlcNAc) in affecting β-lactam susceptibility. Specifically, higher levels of UDP-GlcNAc in H482Y are correlated with CEF sensitivity, which we ascribe to a loss of GlmR-mediated activation of GlmS. Metabolic feeding studies and genetic epistasis suggests that this is a direct cause of the altered resistance. Conversely, the S487L mutant maintains high levels of GlcN-6-P and low levels of UDP-GlcNAc, and, in this strain, GlmR-mediated activation of GlmS is critical for maintaining PG synthesis. Although not the intent of this study, our results have served to highlight the importance of GlmR as a key regulator of metabolic flux through GlmS, the enzyme that shunts carbon from glycolysis/gluconeogenesis into amino sugar and PG synthesis. Drugs that inhibit PG synthesis cause a buildup of cell wall intermediates, including UDP-GlcNAc (67). When UDP-GlcNAc levels increase, it binds to GlmR, and flux into PG synthesis may be reduced. Because GlmR is conserved in many bacteria, including MTB (68, 69), these types of effects are important to consider when examining mechanisms of adaptation and resistance to cell wall antibiotics.

**FIG 8:**
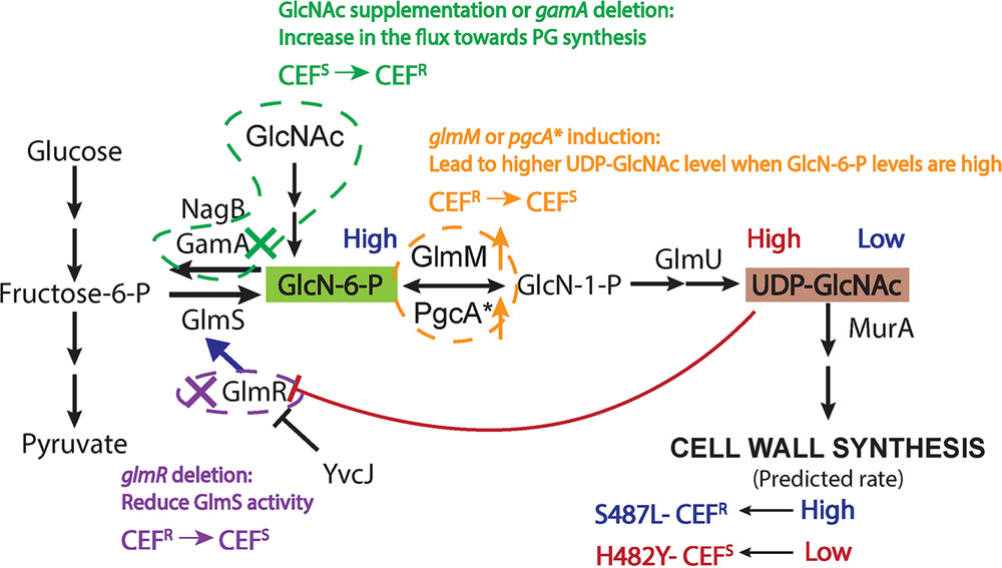
Interpretation of experimental perturbations predicted to affect UDP-GlcNAc levels. The text and arrow in blue summarize the data for the CEF^R^ S487L mutant. This mutant has low levels of UDP-GlcNAc. Thus, GlmR is free to stimulate GlmS activity, and cells are predicted to maintain a high rate of PG synthesis detected by high levels of GlcN-6-P. The text and arrow in red summarize the data for the CEF^S^ H482Y mutant. This mutant has high levels of UDP-GlcNAc, which would bind with GlmR. The bound GlmR is unavailable to stimulate GlmS activity and is thereby predicted to reduce PG synthesis. Three perturbation scenarios are also presented. In green, cells were supplemented with 20 mM GlcNAc or *gamA* was deleted. Both led to higher flux toward PG synthesis independent of GlmS, thereby bypassing the bottleneck in the H482Y mutant and leading to elevated CEF resistance. In orange, induction of *glmM* or pgcA* is predicted to increase the levels of UDP-GlcNAc but only in S487L, which has high levels of GlcN-6-P. Thus, this treatment is predicted to block GlmR-dependent GlmS activation in S487L, reduce PG synthesis, and thereby contribute to CEF sensitivity. These inferences are supported by analysis of the effects of a *glmR* deletion (purple). In S487L, we observed low UDP-GlcNAc levels and predict that GlmR is activating GlmS. Consistently, deletion of *glmR* makes the S487L strain more CEF sensitive. In contrast, in H482Y, we predict that the high observed UDP-GlcNAc levels will keep GlmR sequestered in an inactive state, and consistently there is no effect of deleting *glmR*.

Here, we have validated the central role of GlmR as a regulator and UDP-GlcNAc as a regulatory metabolite using the divergent effects of the S487L and H482Y RRDR mutations on CEF resistance. We have used three experimental perturbations to alter the availability of metabolites to support PG synthesis: (i) GlcNAc supplementation and restriction of catabolism (*gamA* deletion), (ii) elevated expression of *glmM* or pgcA*, and (iii) deletion of *glmR* (Fig. 8). The CEF^R^ S487L mutant maintains high levels of GlcN-6-P and low levels of UDP-GlcNAc. Thus, in this strain, GlmR is active and maintains relatively higher flux of PG synthesis and is better able to tolerate high levels of CEF. As predicted, if GlmR is deleted, the cells become more sensitive to CEF (Fig. 6). Due to the high levels of GlcN-6-P in this strain, induction of *glmM* and pgcA*, combined with elevated *glmU* expression (Fig. 4), may lead to elevation of UDP-GlcNAc. High UDP-GlcNAc, in turn, will inactivate GlmR, restrict flux to PG synthesis, and increase CEF sensitivity (Fig. 7B and 8). In the case of CEF^S^ H482Y, the cells already have high levels of UDP-GlcNAc, which restricts PG synthesis and confers CEF sensitivity. The only manipulation that increased the resistance of this strain was supplementation with GlcNAc or *gamA* deletion, both of which increase PG synthesis independent of GlmR (Fig. 7A and 8).

In summary, we have shown that RIF-resistant RRDR mutants have altered susceptibility to β-lactams due to altered levels of PG metabolites, especially UDP-GlcNAc. RRDR mutations have a global impact on the transcriptome of the cells and can lead to pleiotropic effects. Analyzing the expression levels of PG synthesis genes did not reveal why UDP-GlcNAc levels are altered by RRDR mutations, but the downstream effects of this altered metabolite can account for differences in sensitivity to β-lactams. β-Lactams are some of the most powerful antibiotics and are being considered in TB therapy with RIF (37). Thus, this work on evolution of resistance to the combination of RIF and CEF, the collateral sensitivity to CEF on the acquisition of RIF resistance, and the differential response of *rpoB* mutants to CEF will benefit future studies designing effective drug treatments.

## MATERIALS AND METHODS

### Bacterial strains, plasmids, and growth conditions.

Bacterial strains used in this study are listed in Table S1 in the supplemental material. All stains were grown in lysogeny broth (LB) medium at 37°C. Liquid cultures were aerated on an orbital shaker at 280 rpm. Glycerol stocks were streaked on LB agar plates and incubated overnight at 37°C. *rpoB* was amplified using the primers mentioned in Table S2. Mutations in the RIF resistance-determining region (RRDR) of *rpoB* were confirmed by Sanger sequencing at the Biotechnology Resources core facility at Cornell University using primer 9286. *glmR::erm* and *gamA*::*erm* were ordered from the *Bacillus* knockout erythromycin (BKE) collection available at the *Bacillus* Genetic Stock Centre (BGSC) (70). The gene deletion with the erythromycin cassette was then transformed into the desired strains by natural competence induced in modified competence (MC) medium. The cassette was removed using pDR244 as described previously (70). Transformation was done using chromosomal DNA with selection on plates with 1 μg/mL erythromycin and 25 μg/mL lincomycin. The deletion was confirmed by PCR with check primers listed in Table S2. Strains with inducible expression of *glmM* (HB16910), pgcA* (HB16946), and *pgcA* (HB16945) were made using chromosomal DNA from strains from a previous study (54). Genes were ectopically expressed at the *amyE* locus under promoter P*_spac(hy)_*, and selection of transformants was performed in the presence of chloramphenicol (10 μg/mL).

10.1128/mbio.03168-22.1 TABLE S1 Bacterial strains used in the study. Download Table S1, DOCX file, 0.02 MB. Copyright © 2023 Patel et al. 2023 Patel et al. https://creativecommons.org/licenses/by/4.0/ This content is distributed under the terms of the Creative Commons Attribution 4.0 International license.

10.1128/mbio.03168-22.2 TABLE S2 Primers used in the study. Download Table S2, DOCX file, 0.01 MB. Copyright © 2023 Patel et al. 2023 Patel et al. https://creativecommons.org/licenses/by/4.0/ This content is distributed under the terms of the Creative Commons Attribution 4.0 International license.

### Growth kinetics and MIC determinations.

A Bioscreen C growth curve analyzer (Growth Curves USA, NJ) was used to monitor the growth of the strains. Initially, cultures were grown up to an optical density at 600 nm (OD_600_) of ~0.4 in 5-mL culture tubes. One microliter of this culture was inoculated in each well of honeycomb 100-well plates containing 200 μL of LB medium. The OD_600_ was monitored every 15 min for up to 24 h with constant shaking at 37°C. For MIC determination, 2-fold increases in drug concentrations were screened ranging from 0.04 to 10.24 μg/mL for CEF and from 0.075 to 4 μg/mL for RIF. The minimum concentration of drug having at least 90% growth inhibition compared to the untreated control after 8 h of treatment was considered the drug MIC. Control cells reached stationary phase within 8 h (OD_600_ of ~1.0). Percent inhibition was calculated as

$$\text{\% inhibition}=\left(1\;-\;\left(\frac{{\text{average OD}}_{\text{600}}\;\text{of treated cells}}{{\text{average OD}}_{\text{600}}\;\text{of control cells}}\right)\right)\times 100.$$

Average OD_600_ was calculated from three biological replicates.

### Synergy quantification.

Checkerboard assays were used to determine the interaction between RIF and CEF (71) with 2-fold dilutions of both drugs. One microliter of cultures grown to an OD_600_ of 0.4 was added to each well containing 200 μL of medium with either or both drugs. The MIC of the drug combination was determined as mentioned in the previous section. To quantify the interaction between the two drugs, we calculated both a fractional inhibitory concentration index (FICI) and a ZIP score. The formula to calculate FICI is

$$\text{FICI}=\left(\frac{\text{MIC of drug A in combination}}{\text{MIC of drug A alone}}\right)+\left(\frac{\text{MIC of drug B in combination}}{\text{MIC of drug B alone}}\right).$$

If the value of FICI is ≤0.5, the interaction was considered to be synergistic (72). The ZIP score was calculated using synergy finder (27). A ZIP score of >10 indicates synergy between the two drugs.

### Evolution and whole-genome sequencing.

Wild-type (WT) cells were evolved under the combined treatment of RIF and CEF. Initially, WT cells were grown up to an OD_600_ of 0.4. Twenty-five microliters of these cells was added to 5 mL of LB containing no drug, 0.06 μg/mL RIF with 2.56 μg/mL CEF, 0.12 μg/mL RIF with 2.56 μg/mL CEF, or 0.06 μg/mL RIF with 5.12 μg/mL CEF. The cultures were allowed to grow overnight. The next day, 25 μL of the overnight cultures was transferred to fresh tubes containing 5 mL of LB with the same conditions. This designated the first passage. All cultures were evolved for 10 passages. Cells from each passage were stored as glycerol stocks. For experiments, the frozen stocks were streaked on LB agar plates, and a representative single colony was picked from each passage and analyzed for their RIF and CEF sensitivities. These single colonies were again stored as glycerol stocks. Chromosomal DNA was extracted from the selected single colonies using a Qiagen DNA extraction kit and was sent for whole-genome sequencing. Sequencing was done using the Illumina platform at the Microbial Genome Sequencing Center (MiGS, Pittsburgh). The results were trimmed, mapped, and aligned with reference WT (NC_000964.3) genome sequence using CLC genomics workbench.

### Disk diffusion assay.

Drug susceptibilities of the mutants were screened by determining the zone of inhibition using a disk diffusion assay. Cultures were grown up to an OD_600_ of ~0.4. One hundred microliters of this culture was mixed with 4 mL of top agar (0.75% agar). Top agar was kept at 50°C to prevent it from solidifying. The mix of agar and culture was poured onto a 15-mL LB agar (1.5%) plate. This was allowed to air dry for 30 min. A 6-mm Whatman paper filter disk was then put on the top agar. The required amount of drug was added on the disk immediately. The plates were incubated overnight at 37°C. The diameter of the clear zone of inhibition/low-density growth (ZOI/ZOLD) was measured the next day. For all histograms, the *y* axis starts from 6 mm, which is the disk diameter. For experiments with GlcNAc supplementation, 20 mM GlcNAc was added to both the top agar and LB agar plates. For strains with the inducible promoter P*_spac(hy)_*, the agar was made with 1 mM isopropyl-β-d-thiogalactopyranoside (IPTG). The following amounts of drugs were used on the disks: CEF, 25 μg; RIF, 25 μg; oxacillin, 3 μg; ampicillin, 15 μg; penicillin G, 20 U; nisin, 100 μg; vancomycin, 10 μg; fosfomycin, 75 μg; chloramphenicol, 8 μg; triclosan, 5 μg; paraquat, 8 μL from a 10 mM stock.

### Real-time PCR.

Gene expression was determined by real-time PCR using primers mentioned in Table S2. Cultures were grown up to an OD_600_ of ~0.4. RNA was purified from 1.5 mL of cells using the RNeasy kit from Qiagen as per the manufacturer’s instructions. The isolated RNA was then given a DNase treatment with a Turbo DNA-free kit (Invitrogen, AM1907). Approximately 15 μg of RNA was incubated with 2 μL of DNase and 2 μL of buffer at 37°C for 15 min, followed by a 5-min incubation with the DNase-inactivating agent. The samples were then centrifuged at 8,000 rpm for 3 min, and the supernatant was collected in a fresh microcentrifuge tube. cDNA was prepared with 2 μg of the treated RNA in 20 μL total volume of reaction mix using a high-capacity cDNA reverse transcription kit from Applied Biosystems (4368814). The cDNA was further diluted 1:10 to obtain a final concentration of 10 ng/μL. Gene expression levels were measured using 10 ng of cDNA, 0.5 μM gene specific primers, and 1× SYBR green master mix (Applied Biosystems, A25742) in a StepOnePlus system from Applied Biosystems. *gyrA* was used as an internal control. Gene expression values (2^−Δ^*^CT^*) were plotted after normalization with *gyrA*.

### Metabolite extraction.

Metabolomics experiments were done according to previously published work (73, 74). Both wild-type and mutant strains were first grown in 5 mL of LB broth (BD Difco) medium at 30°C for 12 h and were diluted 1:50 in 40 mL of medium (in triplicates) and grown at 37°C. Mid-log-phase cultures with an OD_600_ of 0.4 were pelleted and quenched by resuspending in 700 μL of a precooled 40%:40%:20% mixture of acetonitrile, methanol, and water. To extract metabolites, cells were lysed using 0.1-mm Zirconia beads and a Precellys homogenizer (Bertin Instruments). Lysates were centrifuged at 12,000 rpm for 8 min at 37°C and cleared by passing through 0.22-μm Spin-X tube filters (Sigma-Aldrich).

### Liquid chromatography and mass spectrometry.

Two microliters of extracted metabolite samples was separated on a Cogent Diamond Hydride type C column of 1200 liquid chromatography (Agilent), which was coupled to an Agilent accurate mass 6220 time of flight spectrometer. For different classes of metabolites, two types of solvents were used: (i) solvent A (water + 0.2% formic acid) and (ii) solvent B (acetonitrile + 0.2% formic acid). The gradient was 0 to 2 min, 85% B; 3 to 5 min, 80% B; 6 to 7 min, 75% B; 8 to 9 min, 70% B; 10 to 11.1 min, 50% B; 11.1 to 14 min, 20% B; and 14.1 to 24 min, 5% B, with a 10-min reequilibration period at 85% B at a flow rate of 0.4 mL/min. For dynamic mass axis calibration, a reference mass solution was continuously injected from the isocratic pump. Ion abundances of different metabolites were determined using Profinder 8.0. The log_2_ fold change values were calculated with respect to the abundances in the wild-type strain.

### Statistical analysis.

All experiments were performed with a minimum of three biological replicates. One-way analysis of variance (ANOVA) was used to calculate the statistical significance. A Tukey’s comparison test was used to determine significance between all the strains. *P* value cutoffs are mentioned in the figure legends. Different letters represent data that are significantly different. Same letters represent mean values that are not statistically different. Significance between two strains was determined using a Student’s *t* test.

## References

[1] Storz G, Hengge R. 2011. Bacterial stress responses, 2nd ed. ASM Press, Washington, DC.

[2] Foster PL. 2007. Stress-induced mutagenesis in bacteria. Crit Rev Biochem Mol Biol 42:373–397. doi:10.1080/10409230701648494. 17917873 PMC2747772

[3] Cohen Y, Hershberg R. 2022. Rapid adaptation often occurs through mutations to the most highly conserved positions of the RNA polymerase core enzyme. Genome Biol Evol 14:evac105. doi:10.1093/gbe/evac105. 35876137 PMC9459352

[4] LaCroix RA, Sandberg TE, O'Brien EJ, Utrilla J, Ebrahim A, Guzman GI, Szubin R, Palsson BO, Feist AM. 2015. Use of adaptive laboratory evolution to discover key mutations enabling rapid growth of *Escherichia coli* K-12 MG1655 on glucose minimal medium. Appl Environ Microbiol 81:17–30. doi:10.1128/AEM.02246-14. 25304508 PMC4272732

[5] Kuehne SA, Dempster AW, Collery MM, Joshi N, Jowett J, Kelly ML, Cave R, Longshaw CM, Minton NP. 2018. Characterization of the impact of *rpoB* mutations on the *in vitro* and *in vivo* competitive fitness of *Clostridium difficile* and susceptibility to fidaxomicin. J Antimicrob Chemother 73:973–980. doi:10.1093/jac/dkx486. 29253242 PMC5890677

[6] Panchal VV, Griffiths C, Mosaei H, Bilyk B, Sutton JAF, Carnell OT, Hornby DP, Green J, Hobbs JK, Kelley WL, Zenkin N, Foster SJ. 2020. Evolving MRSA: high-level beta-lactam resistance in *Staphylococcus aureus* is associated with RNA polymerase alterations and fine tuning of gene expression. PLoS Pathog 16:e1008672. doi:10.1371/journal.ppat.1008672. 32706832 PMC7380596

[7] Shiver AL, Osadnik H, Peters JM, Mooney RA, Wu PI, Henry KK, Braberg H, Krogan NJ, Hu JC, Landick R, Huang KC, Gross CA. 2021. Chemical-genetic interrogation of RNA polymerase mutants reveals structure-function relationships and physiological tradeoffs. Mol Cell 81:2201–2215. doi:10.1016/j.molcel.2021.04.027. 34019789 PMC8484514

[8] Ramaswamy S, Musser JM. 1998. Molecular genetic basis of antimicrobial agent resistance in *Mycobacterium tuberculosis*: 1998 update. Tuber Lung Dis 79:3–29. doi:10.1054/tuld.1998.0002. 10645439

[9] Molodtsov V, Scharf NT, Stefan MA, Garcia GA, Murakami KS. 2017. Structural basis for rifamycin resistance of bacterial RNA polymerase by the three most clinically important RpoB mutations found in *Mycobacterium tuberculosis*. Mol Microbiol 103:1034–1045. doi:10.1111/mmi.13606. 28009073 PMC5344776

[10] Koch A, Mizrahi V, Warner DF. 2014. The impact of drug resistance on *Mycobacterium tuberculosis* physiology: what can we learn from rifampicin? Emerg Microbes Infect 3:e17. doi:10.1038/emi.2014.17. 26038512 PMC3975073

[11] Mariam DH, Mengistu Y, Hoffner SE, Andersson DI. 2004. Effect of *rpoB* mutations conferring rifampin resistance on fitness of *Mycobacterium tuberculosis*. Antimicrob Agents Chemother 48:1289–1294. doi:10.1128/AAC.48.4.1289-1294.2004. 15047531 PMC375340

[12] Xu M, Zhou YN, Goldstein BP, Jin DJ. 2005. Cross-resistance of *Escherichia coli* RNA polymerases conferring rifampin resistance to different antibiotics. J Bacteriol 187:2783–2792. doi:10.1128/JB.187.8.2783-2792.2005. 15805525 PMC1070395

[13] Lahiri N, Shah RR, Layre E, Young D, Ford C, Murray MB, Fortune SM, Moody DB. 2016. Rifampin resistance mutations are associated with broad chemical remodeling of *Mycobacterium tuberculosis*. J Biol Chem 291:14248–14256. doi:10.1074/jbc.M116.716704. 27226566 PMC4933180

[14] Sonenshein AL, Losick R. 1970. RNA polymerase mutants blocked in sporulation. Nature 227:906–909. doi:10.1038/227906a0. 4988656

[15] Perkins AE, Nicholson WL. 2008. Uncovering new metabolic capabilities of *Bacillus subtilis* using phenotype profiling of rifampin-resistant *rpoB* mutants. J Bacteriol 190:807–814. doi:10.1128/JB.00901-07. 17644585 PMC2223569

[16] Campodonico VL, Rifat D, Chuang YM, Ioerger TR, Karakousis PC. 2018. Altered *Mycobacterium tuberculosis* cell wall metabolism and physiology associated with RpoB mutation H526D. Front Microbiol 9:494. doi:10.3389/fmicb.2018.00494. 29616007 PMC5867343

[17] Giddey AD, Ganief TA, Ganief N, Koch A, Warner DF, Soares NC, Blackburn JM. 2021. Cell wall proteomics reveal phenotypic adaption of drug-resistant *Mycobacterium smegmatis* to subinhibitory rifampicin exposure. Front Med (Lausanne) 8:723667. doi:10.3389/fmed.2021.723667. 34676224 PMC8525676

[18] Fajardo-Cavazos P, Leehan JD, Nicholson WL. 2018. Alterations in the spectrum of spontaneous rifampicin-resistance mutations in the *Bacillus subtilis rpoB* gene after cultivation in the human spaceflight environment. Front Microbiol 9:192. doi:10.3389/fmicb.2018.00192. 29491852 PMC5817088

[19] Leehan JD, Nicholson WL. 2021. The spectrum of spontaneous rifampin resistance mutations in the *Bacillus subtilis rpoB* gene depends on the growth environment. Appl Environ Microbiol 87:e0123721. doi:10.1128/AEM.01237-21. 34495706 PMC8552901

[20] Leehan JD, Nicholson WL. 2022. Environmental dependence of competitive fitness in rifampin-resistant *rpoB* mutants of *Bacillus subtilis*. Appl Environ Microbiol 88:e0242221. doi:10.1128/aem.02422-21. 35258334 PMC8904044

[21] Sotgiu G, Centis R, D'Ambrosio L, Migliori GB. 2015. Tuberculosis treatment and drug regimens. Cold Spring Harb Perspect Med 5:a017822. doi:10.1101/cshperspect.a017822. 25573773 PMC4448591

[22] Gold B, Zhang J, Quezada LL, Roberts J, Ling Y, Wood M, Shinwari W, Goullieux L, Roubert C, Fraisse L, Bacque E, Lagrange S, Filoche-Romme B, Vieth M, Hipskind PA, Jungheim LN, Aube J, Scarry SM, McDonald SL, Li K, Perkowski A, Nguyen Q, Dartois V, Zimmerman M, Olsen DB, Young K, Bonnett S, Joerss D, Parish T, Boshoff HI, Arora K, Barry CE, III, Guijarro L, Anca S, Rullas J, Rodriguez-Salguero B, Martinez-Martinez MS, Porras-De Francisco E, Cacho M, Barros-Aguirre D, Smith P, Berthel SJ, Nathan C, Bates RH. 2022. Identification of beta-lactams active against *Mycobacterium tuberculosis* by a consortium of pharmaceutical companies and academic institutions. ACS Infect Dis 8:557–573. doi:10.1021/acsinfecdis.1c00570. 35192346 PMC8922279

[23] Sharifzadeh S, Dempwolff F, Kearns DB, Carlson EE. 2020. Harnessing beta-lactam antibiotics for illumination of the activity of penicillin-binding proteins in *Bacillus subtilis*. ACS Chem Biol 15:1242–1251. doi:10.1021/acschembio.9b00977. 32155044 PMC7784776

[24] Brandt CM, Rouse MS, Tallan BM, Laue NW, Wilson WR, Steckelberg JM. 1995. Effective treatment of cephalosporin-rifampin combinations against cryptic methicillin-resistant beta-lactamase-producing coagulase-negative staphylococcal experimental endocarditis. Antimicrob Agents Chemother 39:1815–1819. doi:10.1128/AAC.39.8.1815. 7486924 PMC162831

[25] Kaushik A, Makkar N, Pandey P, Parrish N, Singh U, Lamichhane G. 2015. Carbapenems and rifampin exhibit synergy against *Mycobacterium tuberculosis* and *Mycobacterium abscessus*. Antimicrob Agents Chemother 59:6561–6567. doi:10.1128/AAC.01158-15. 26259792 PMC4576034

[26] Patel Y, Zhao H, Helmann JD. 2020. A regulatory pathway that selectively up-regulates elongasome function in the absence of class A PBPs. eLife 9:e57902. doi:10.7554/eLife.57902. 32897856 PMC7478892

[27] Yadav B, Wennerberg K, Aittokallio T, Tang J. 2015. Searching for drug synergy in complex dose-response landscapes using an interaction potency model. Comput Struct Biotechnol J 13:504–513. doi:10.1016/j.csbj.2015.09.001. 26949479 PMC4759128

[28] Konate K, Mavoungou JF, Lepengue AN, Aworet-Samseny RR, Hilou A, Souza A, Dicko MH, M'Batchi B. 2012. Antibacterial activity against beta-lactamase producing methicillin and ampicillin-resistants *Staphylococcus aureus*: fractional inhibitory concentration index (FICI) determination. Ann Clin Microbiol Antimicrob 11:18. doi:10.1186/1476-0711-11-18. 22716026 PMC3464800

[29] Michel JB, Yeh PJ, Chait R, Moellering RC, Jr, Kishony R. 2008. Drug interactions modulate the potential for evolution of resistance. Proc Natl Acad Sci USA 105:14918–14923. doi:10.1073/pnas.0800944105. 18815368 PMC2567468

[30] Aiba Y, Katayama Y, Hishinuma T, Murakami-Kuroda H, Cui L, Hiramatsu K. 2013. Mutation of RNA polymerase beta-subunit gene promotes heterogeneous-to-homogeneous conversion of beta-lactam resistance in methicillin-resistant *Staphylococcus aureus*. Antimicrob Agents Chemother 57:4861–4871. doi:10.1128/AAC.00720-13. 23877693 PMC3811421

[31] Jin DJ, Gross CA. 1988. Mapping and sequencing of mutations in the *Escherichia coli rpoB* gene that lead to rifampicin resistance. J Mol Biol 202:45–58. doi:10.1016/0022-2836(88)90517-7. 3050121

[32] Vogler AJ, Busch JD, Percy-Fine S, Tipton-Hunton C, Smith KL, Keim P. 2002. Molecular analysis of rifampin resistance in *Bacillus anthracis* and *Bacillus cereus*. Antimicrob Agents Chemother 46:511–513. doi:10.1128/AAC.46.2.511-513.2002. 11796364 PMC127050

[33] Muthaiah M, Shivekar SS, Cuppusamy Kapalamurthy VR, Alagappan C, Sakkaravarthy A, Brammachary U. 2017. Prevalence of mutations in genes associated with rifampicin and isoniazid resistance in *Mycobacterium tuberculosis* clinical isolates. J Clin Tuberc Other Mycobact Dis 8:19–25. doi:10.1016/j.jctube.2017.06.001. 31723707 PMC6850230

[34] Hauck Y, Fabre M, Vergnaud G, Soler C, Pourcel C. 2009. Comparison of two commercial assays for the characterization of *rpoB* mutations in *Mycobacterium tuberculosis* and description of new mutations conferring weak resistance to rifampicin. J Antimicrob Chemother 64:259–262. doi:10.1093/jac/dkp204. 19520715

[35] Nicholson WL, Maughan H. 2002. The spectrum of spontaneous rifampin resistance mutations in the *rpoB* gene of *Bacillus subtilis* 168 spores differs from that of vegetative cells and resembles that of *Mycobacterium tuberculosis*. J Bacteriol 184:4936–4940. doi:10.1128/JB.184.17.4936-4940.2002. 12169622 PMC135274

[36] Li MC, Lu J, Lu Y, Xiao TY, Liu HC, Lin SQ, Xu D, Li GL, Zhao XQ, Liu ZG, Zhao LL, Wan KL. 2021. *rpoB* mutations and effects on rifampin resistance in *Mycobacterium tuberculosis*. Infect Drug Resist 14:4119–4128. doi:10.2147/IDR.S333433. 34675557 PMC8502021

[37] De Jager V, Gupte N, Nunes S, Barnes GL, van Wijk RC, Mostert J, Dorman SE, Abulfathi AA, Upton CM, Faraj A, Nuermberger EL, Lamichhane G, Svensson EM, Simonsson USH, Diacon AH, Dooley KE. 2022. Early bactericidal activity of meropenem plus clavulanate (with or without rifampin) for tuberculosis: the COMRADE randomized, phase 2A clinical trial. Am J Respir Crit Care Med 205:1228–1235. doi:10.1164/rccm.202108-1976OC. 35258443 PMC9872811

[38] Beckley AM, Wright ES. 2021. Identification of antibiotic pairs that evade concurrent resistance via a retrospective analysis of antimicrobial susceptibility test results. Lancet Microbe 2:e545–e554. doi:10.1016/S2666-5247(21)00118-X. 34632433 PMC8496867

[39] Watanakunakorn C. 1984. Mode of action and *in vitro* activity of vancomycin. J Antimicrob Chemother 14 Suppl D:7–18. doi:10.1093/jac/14.suppl_D.7. 6440886

[40] Wiedemann I, Breukink E, van Kraaij C, Kuipers OP, Bierbaum G, de Kruijff B, Sahl HG. 2001. Specific binding of nisin to the peptidoglycan precursor lipid II combines pore formation and inhibition of cell wall biosynthesis for potent antibiotic activity. J Biol Chem 276:1772–1779. doi:10.1074/jbc.M006770200. 11038353

[41] Silver LL. 2017. Fosfomycin: mechanism and resistance. Cold Spring Harb Perspect Med 7:a025262. doi:10.1101/cshperspect.a025262. 28062557 PMC5287057

[42] Schlunzen F, Zarivach R, Harms J, Bashan A, Tocilj A, Albrecht R, Yonath A, Franceschi F. 2001. Structural basis for the interaction of antibiotics with the peptidyl transferase centre in eubacteria. Nature 413:814–821. doi:10.1038/35101544. 11677599

[43] Heath RJ, Rubin JR, Holland DR, Zhang E, Snow ME, Rock CO. 1999. Mechanism of triclosan inhibition of bacterial fatty acid synthesis. J Biol Chem 274:11110–11114. doi:10.1074/jbc.274.16.11110. 10196195

[44] Carr RJ, Bilton RF, Atkinson T. 1986. Toxicity of paraquat to microorganisms. Appl Environ Microbiol 52:1112–1116. doi:10.1128/aem.52.5.1112-1116.1986. 3098166 PMC239182

[45] Collins JA, Irnov I, Baker S, Winkler WC. 2007. Mechanism of mRNA destabilization by the *glmS* ribozyme. Genes Dev 21:3356–3368. doi:10.1101/gad.1605307. 18079181 PMC2113035

[46] Winkler WC, Nahvi A, Roth A, Collins JA, Breaker RR. 2004. Control of gene expression by a natural metabolite-responsive ribozyme. Nature 428:281–286. doi:10.1038/nature02362. 15029187

[47] Gaugue I, Oberto J, Plumbridge J. 2014. Regulation of amino sugar utilization in *Bacillus subtilis* by the GntR family regulators, NagR and GamR. Mol Microbiol 92:100–115. doi:10.1111/mmi.12544. 24673833

[48] Bertram R, Rigali S, Wood N, Lulko AT, Kuipers OP, Titgemeyer F. 2011. Regulon of the *N*-acetylglucosamine utilization regulator NagR in *Bacillus subtilis*. J Bacteriol 193:3525–3536. doi:10.1128/JB.00264-11. 21602348 PMC3133301

[49] McCarthy TJ, Plog MA, Floy SA, Jansen JA, Soukup JK, Soukup GA. 2005. Ligand requirements for *glmS* ribozyme self-cleavage. Chem Biol 12:1221–1226. doi:10.1016/j.chembiol.2005.09.006. 16298301

[50] Patel V, Wu Q, Chandrangsu P, Helmann JD. 2018. A metabolic checkpoint protein GlmR is important for diverting carbon into peptidoglycan biosynthesis in *Bacillus subtilis*. PLoS Genet 14:e1007689. doi:10.1371/journal.pgen.1007689. 30248093 PMC6171935

[51] Foulquier E, Pompeo F, Byrne D, Fierobe HP, Galinier A. 2020. Uridine diphosphate *N*-acetylglucosamine orchestrates the interaction of GlmR with either YvcJ or GlmS in *Bacillus subtilis*. Sci Rep 10:15938. doi:10.1038/s41598-020-72854-2. 32994436 PMC7525490

[52] Zhu Y, Eiteman MA, Altman R, Altman E. 2008. High glycolytic flux improves pyruvate production by a metabolically engineered *Escherichia coli* strain. Appl Environ Microbiol 74:6649–6655. doi:10.1128/AEM.01610-08. 18806005 PMC2576684

[53] Alvarez-Anorve LI, Gaugue I, Link H, Marcos-Viquez J, Diaz-Jimenez DM, Zonszein S, Bustos-Jaimes I, Schmitz-Afonso I, Calcagno ML, Plumbridge J. 2016. Allosteric activation of *Escherichia coli* glucosamine-6-phosphate deaminase (NagB) *in vivo* justified by intracellular amino sugar metabolite concentrations. J Bacteriol 198:1610–1620. doi:10.1128/JB.00870-15. 27002132 PMC4959280

[54] Patel V, Black KA, Rhee KY, Helmann JD. 2019. *Bacillus subtilis* PgcA moonlights as a phosphoglucosamine mutase in support of peptidoglycan synthesis. PLoS Genet 15:e1008434. doi:10.1371/journal.pgen.1008434. 31589605 PMC6797236

[55] Bollenbach T. 2015. Antimicrobial interactions: mechanisms and implications for drug discovery and resistance evolution. Curr Opin Microbiol 27:1–9. doi:10.1016/j.mib.2015.05.008. 26042389

[56] Arenaz-Callao MP, Gonzalez Del Rio R, Lucia Quintana A, Thompson CJ, Mendoza-Losana A, Ramon-Garcia S. 2019. Triple oral beta-lactam containing therapy for Buruli ulcer treatment shortening. PLoS Negl Trop Dis 13:e0007126. doi:10.1371/journal.pntd.0007126. 30689630 PMC6366712

[57] Jiang Z, He X, Li J. 2018. Synergy effect of meropenem-based combinations against *Acinetobacter baumannii*: a systematic review and meta-analysis. Infect Drug Resist 11:1083–1095. doi:10.2147/IDR.S172137. 30122965 PMC6086107

[58] Ramon-Garcia S, Gonzalez Del Rio R, Villarejo AS, Sweet GD, Cunningham F, Barros D, Ballell L, Mendoza-Losana A, Ferrer-Bazaga S, Thompson CJ. 2016. Repurposing clinically approved cephalosporins for tuberculosis therapy. Sci Rep 6:34293. doi:10.1038/srep34293. 27678056 PMC5039641

[59] Tangden T, Hickman RA, Forsberg P, Lagerback P, Giske CG, Cars O. 2014. Evaluation of double- and triple-antibiotic combinations for VIM- and NDM-producing *Klebsiella pneumoniae* by *in vitro* time-kill experiments. Antimicrob Agents Chemother 58:1757–1762. doi:10.1128/AAC.00741-13. 24395223 PMC3957864

[60] Rodriguez de Evgrafov M, Gumpert H, Munck C, Thomsen TT, Sommer MO. 2015. Collateral resistance and sensitivity modulate evolution of high-level resistance to drug combination treatment in *Staphylococcus aureus*. Mol Biol Evol 32:1175–1185. doi:10.1093/molbev/msv006. 25618457

[61] Cai XC, Xi H, Liang L, Liu JD, Liu CH, Xue YR, Yu XY. 2017. Rifampicin-resistance mutations in the *rpoB* gene in *Bacillus velezensis* CC09 have pleiotropic effects. Front Microbiol 8:178. doi:10.3389/fmicb.2017.00178. 28243227 PMC5303731

[62] Rifat D, Campodonico VL, Tao J, Miller JA, Alp A, Yao Y, Karakousis PC. 2017. *In vitro* and *in vivo* fitness costs associated with *Mycobacterium tuberculosis* RpoB mutation H526D. Future Microbiol 12:753–765. doi:10.2217/fmb-2017-0022. 28343421 PMC5506874

[63] Srivastava A, Degen D, Ebright YW, Ebright RH. 2012. Frequency, spectrum, and nonzero fitness costs of resistance to myxopyronin in *Staphylococcus aureus*. Antimicrob Agents Chemother 56:6250–6255. doi:10.1128/AAC.01060-12. 23006749 PMC3497154

[64] Kurepina N, Chudaev M, Kreiswirth BN, Nikiforov V, Mustaev A. 2022. Mutations compensating for the fitness cost of rifampicin resistance in *Escherichia coli* exert pleiotropic effect on RNA polymerase catalysis. Nucleic Acids Res 50:5739–5756. doi:10.1093/nar/gkac406. 35639764 PMC9177976

[65] Cui L, Isii T, Fukuda M, Ochiai T, Neoh HM, Camargo IL, Watanabe Y, Shoji M, Hishinuma T, Hiramatsu K. 2010. An RpoB mutation confers dual heteroresistance to daptomycin and vancomycin in *Staphylococcus aureus*. Antimicrob Agents Chemother 54:5222–5233. doi:10.1128/AAC.00437-10. 20837752 PMC2981288

[66] Rasouly A, Shamovsky Y, Epshtein V, Tam K, Vasilyev N, Hao Z, Quarta G, Pani B, Li L, Vallin C, Shamovsky I, Krishnamurthy S, Shtilerman A, Vantine S, Torres VJ, Nudler E. 2021. Analysing the fitness cost of antibiotic resistance to identify targets for combination antimicrobials. Nat Microbiol 6:1410–1423. doi:10.1038/s41564-021-00973-1. 34697460 PMC9389595

[67] Lobritz MA, Andrews IW, Braff D, Porter CBM, Gutierrez A, Furuta Y, Cortes LBG, Ferrante T, Bening SC, Wong F, Gruber C, Bakerlee CW, Lambert G, Walker GC, Dwyer DJ, Collins JJ. 2021. Increased energy demand from anabolic-catabolic processes drives beta-lactam antibiotic lethality. Cell Chem Biol 29:276–286. doi:10.1016/j.chembiol.2021.12.010. PMC8857051 34990601

[68] Mir M, Prisic S, Kang CM, Lun S, Guo H, Murry JP, Rubin EJ, Husson RN. 2014. Mycobacterial gene *cuvA* is required for optimal nutrient utilization and virulence. Infect Immun 82:4104–4117. doi:10.1128/IAI.02207-14. 25047842 PMC4187881

[69] Pensinger DA, Boldon KM, Chen GY, Vincent WJ, Sherman K, Xiong M, Schaenzer AJ, Forster ER, Coers J, Striker R, Sauer JD. 2016. The *Listeria monocytogenes* PASTA kinase PrkA and its substrate YvcK are required for cell wall homeostasis, metabolism, and virulence. PLoS Pathog 12:e1006001. doi:10.1371/journal.ppat.1006001. 27806131 PMC5091766

[70] Koo BM, Kritikos G, Farelli JD, Todor H, Tong K, Kimsey H, Wapinski I, Galardini M, Cabal A, Peters JM, Hachmann AB, Rudner DZ, Allen KN, Typas A, Gross CA. 2017. Construction and analysis of two genome-scale deletion libraries for *Bacillus subtilis*. Cell Syst 4:291–305. doi:10.1016/j.cels.2016.12.013. 28189581 PMC5400513

[71] Hsieh MH, Yu CM, Yu VL, Chow JW. 1993. Synergy assessed by checkerboard. A critical analysis. Diagn Microbiol Infect Dis 16:343–349. doi:10.1016/0732-8893(93)90087-n. 8495592

[72] Odds FC. 2003. Synergy, antagonism, and what the chequerboard puts between them. J Antimicrob Chemother 52:1. doi:10.1093/jac/dkg301. 12805255

[73] Planck KA, Rhee K. 2021. Metabolomics of *Mycobacterium tuberculosis*. Methods Mol Biol 2314:579–593. doi:10.1007/978-1-0716-1460-0_25. 34235671

[74] Wang Z, Soni V, Marriner G, Kaneko T, Boshoff HIM, Barry CE, III, Rhee KY. 2019. Mode-of-action profiling reveals glutamine synthetase as a collateral metabolic vulnerability of *M. tuberculosis* to bedaquiline. Proc Natl Acad Sci USA 116:19646–19651. doi:10.1073/pnas.1907946116. 31501323 PMC6765305

