# Supplementary material for: Mutations in rpoB That Confer Rifampicin Resistance Can Alter Levels of Peptidoglycan Precursors and Affect β-Lactam Susceptibility
Source: mBio. 2023 Feb 13;14(2):e03168-22. doi: 10.1128/mbio.03168-22 (PMC10128067; doi:10.1128/mbio.03168-22)
Supplement: TABLE S3 [file mbio.03168-22-s0008.docx]

**Supplementary Table 3** Synergy determination between RIF and CEF by ZIP score

| **CEF (μg/mL)** | **RIF (μg/mL)** | **ZIP score** |
| --- | --- | --- |
| 0 | 0 | 0.00 |
| 0 | 0.0075 | 0.00 |
| 0 | 0.015 | 0.00 |
| 0 | 0.03 | 0.00 |
| 0 | 0.06 | 0.00 |
| 0 | 0.125 | 0.00 |
| 0 | 0.25 | 0.00 |
| 0 | 0.5 | 0.00 |
| 0 | 1 | 0.00 |
| 0 | 2 | 0.00 |
| 0.04 | 0 | 0.00 |
| 0.04 | 0.0075 | **14.43** |
| 0.04 | 0.015 | **20.23** |
| 0.04 | 0.03 | **25.75** |
| 0.04 | 0.06 | **61.68** |
| 0.04 | 0.125 | **36.48** |
| 0.04 | 0.25 | 6.83 |
| 0.04 | 0.5 | -1.07 |
| 0.04 | 1 | -1.55 |
| 0.04 | 2 | -2.91 |
| 0.08 | 0 | 0.00 |
| 0.08 | 0.0075 | **16.41** |
| 0.08 | 0.015 | **24.05** |
| 0.08 | 0.03 | **34.72** |
| 0.08 | 0.06 | **69.12** |
| 0.08 | 0.125 | **36.07** |
| 0.08 | 0.25 | 7.31 |
| 0.08 | 0.5 | -0.29 |
| 0.08 | 1 | -0.74 |
| 0.08 | 2 | -2.10 |
| 0.16 | 0 | 0.00 |
| 0.16 | 0.0075 | 14.88 |
| 0.16 | 0.015 | 22.77 |
| 0.16 | 0.03 | 36.11 |
| 0.16 | 0.06 | 65.66 |
| 0.16 | 0.125 | 33.41 |
| 0.16 | 0.25 | 6.57 |
| 0.16 | 0.5 | -0.46 |
| 0.16 | 1 | -0.88 |
| 0.16 | 2 | -2.23 |
| 0.32 | 0 | 0.00 |
| 0.32 | 0.0075 | 8.93 |
| 0.32 | 0.015 | 15.95 |
| 0.32 | 0.03 | 33.93 |
| 0.32 | 0.06 | 55.81 |
| 0.32 | 0.125 | 27.66 |
| 0.32 | 0.25 | 4.42 |
| 0.32 | 0.5 | -1.54 |
| 0.32 | 1 | -2.06 |
| 0.32 | 2 | -3.39 |
| 0.64 | 0 | 0.00 |
| 0.64 | 0.0075 | 3.89 |
| 0.64 | 0.015 | 10.36 |
| 0.64 | 0.03 | 35.58 |
| 0.64 | 0.06 | 43.14 |
| 0.64 | 0.125 | 20.19 |
| 0.64 | 0.25 | 2.24 |
| 0.64 | 0.5 | -1.83 |
| 0.64 | 1 | -2.91 |
| 0.64 | 2 | -3.00 |
| 1.28 | 0 | 0.00 |
| 1.28 | 0.0075 | 2.31 |
| 1.28 | 0.015 | 3.88 |
| 1.28 | 0.03 | 24.21 |
| 1.28 | 0.06 | 27.61 |
| 1.28 | 0.125 | 12.03 |
| 1.28 | 0.25 | -0.12 |
| 1.28 | 0.5 | -2.70 |
| 1.28 | 1 | -3.57 |
| 1.28 | 2 | -3.82 |
| 2.56 | 0 | 0.00 |
| 2.56 | 0.0075 | 4.44 |
| 2.56 | 0.015 | 4.89 |
| 2.56 | 0.03 | 11.85 |
| 2.56 | 0.06 | 14.26 |
| 2.56 | 0.125 | 4.99 |
| 2.56 | 0.25 | -2.26 |
| 2.56 | 0.5 | -3.81 |
| 2.56 | 1 | -4.26 |
| 2.56 | 2 | -4.75 |
| 5.12 | 0 | 0.00 |
| 5.12 | 0.0075 | 4.36 |
| 5.12 | 0.015 | 5.03 |
| 5.12 | 0.03 | 4.64 |
| 5.12 | 0.06 | 5.20 |
| 5.12 | 0.125 | 0.19 |
| 5.12 | 0.25 | -3.71 |
| 5.12 | 0.5 | -4.65 |
| 5.12 | 1 | -5.07 |
| 5.12 | 2 | -5.69 |
| 10.24 | 0 | 0.00 |
| 10.24 | 0.0075 | -1.54 |
| 10.24 | 0.015 | -0.77 |
| 10.24 | 0.03 | -1.22 |
| 10.24 | 0.06 | -0.01 |
| 10.24 | 0.125 | -2.54 |
| 10.24 | 0.25 | -4.36 |
| 10.24 | 0.5 | -5.00 |
| 10.24 | 1 | -5.37 |
| 10.24 | 2 | -5.99 |
