# Supplementary figures and images for: Mutations in rpoB That Confer Rifampicin Resistance Can Alter Levels of Peptidoglycan Precursors and Affect β-Lactam Susceptibility
Source: mBio. 2023 Feb 13;14(2):e03168-22. doi: 10.1128/mbio.03168-22 (PMC10128067; doi:10.1128/mbio.03168-22)

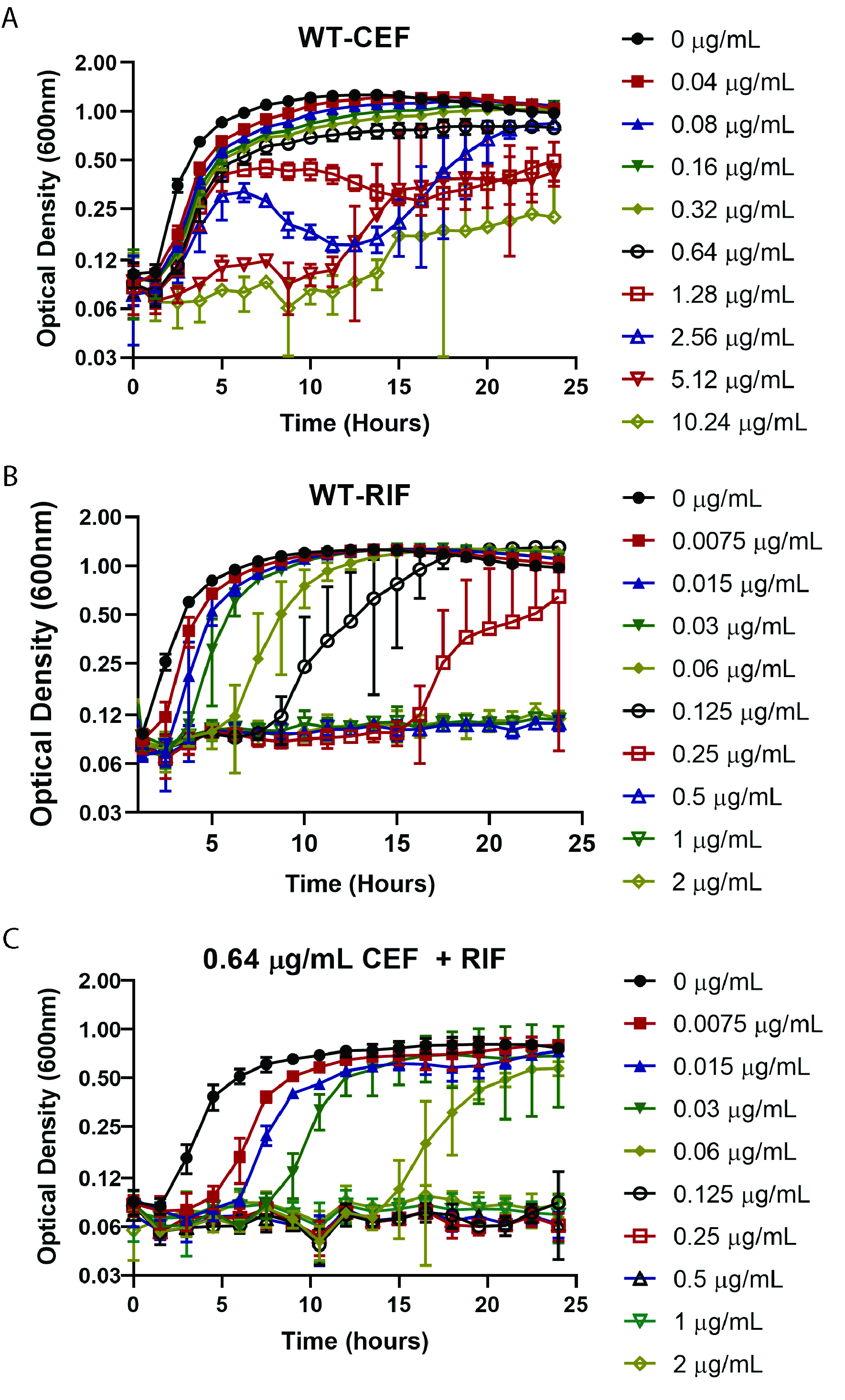

Supplement: FIG S1 [file mbio.03168-22-s0001.tif]

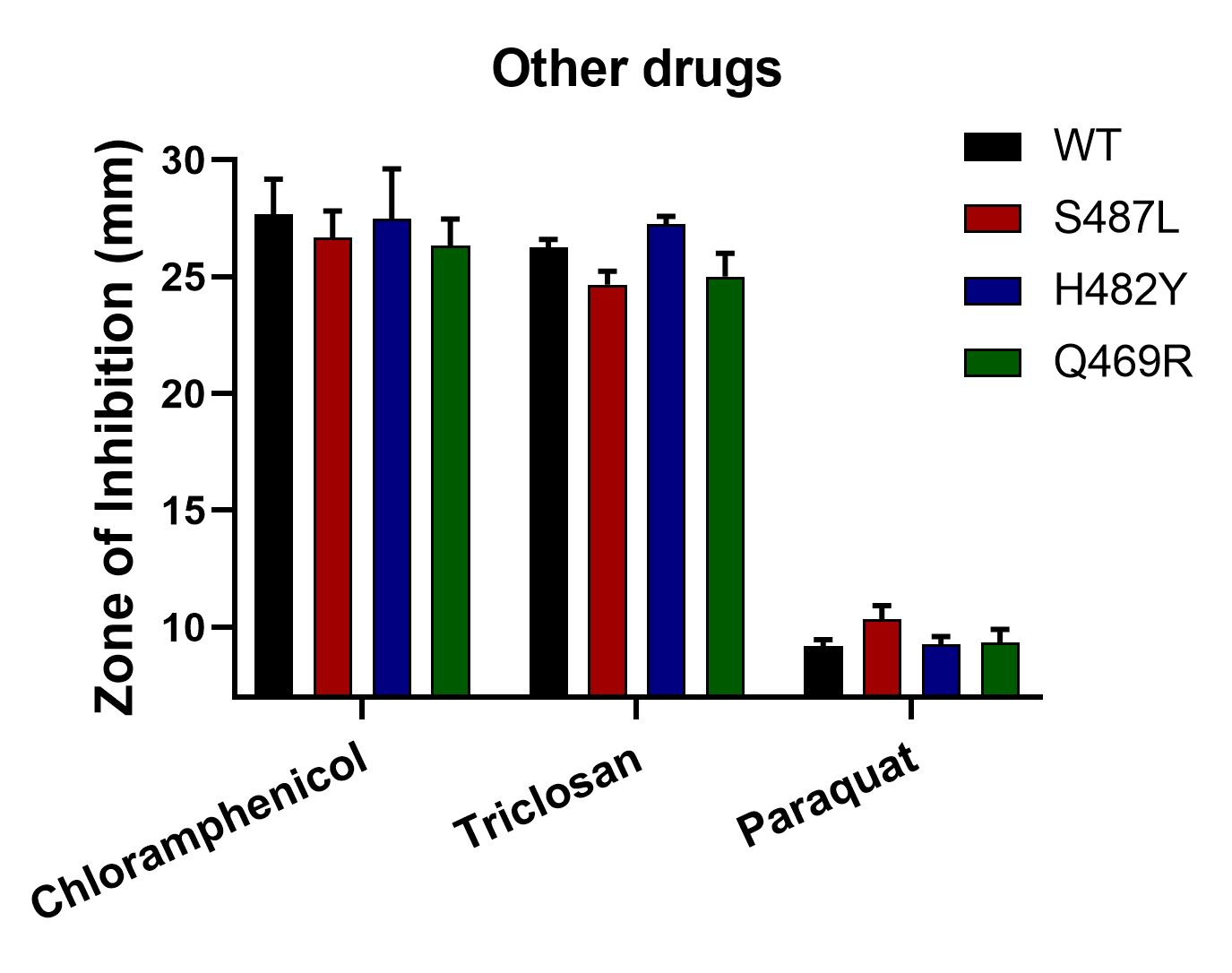

Supplement: FIG S2 [file mbio.03168-22-s0002.tif]

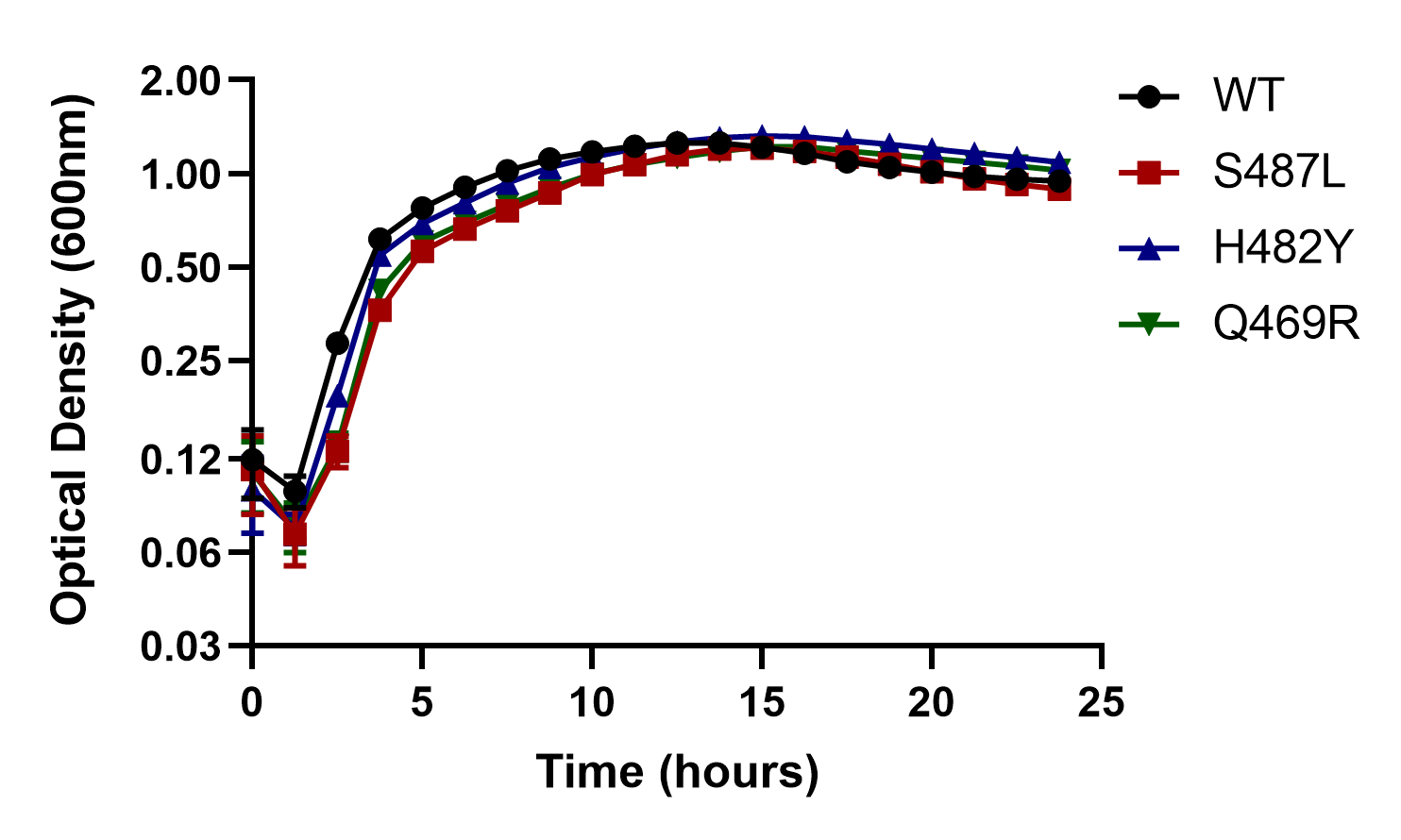

Supplement: FIG S3 [file mbio.03168-22-s0003.tif]

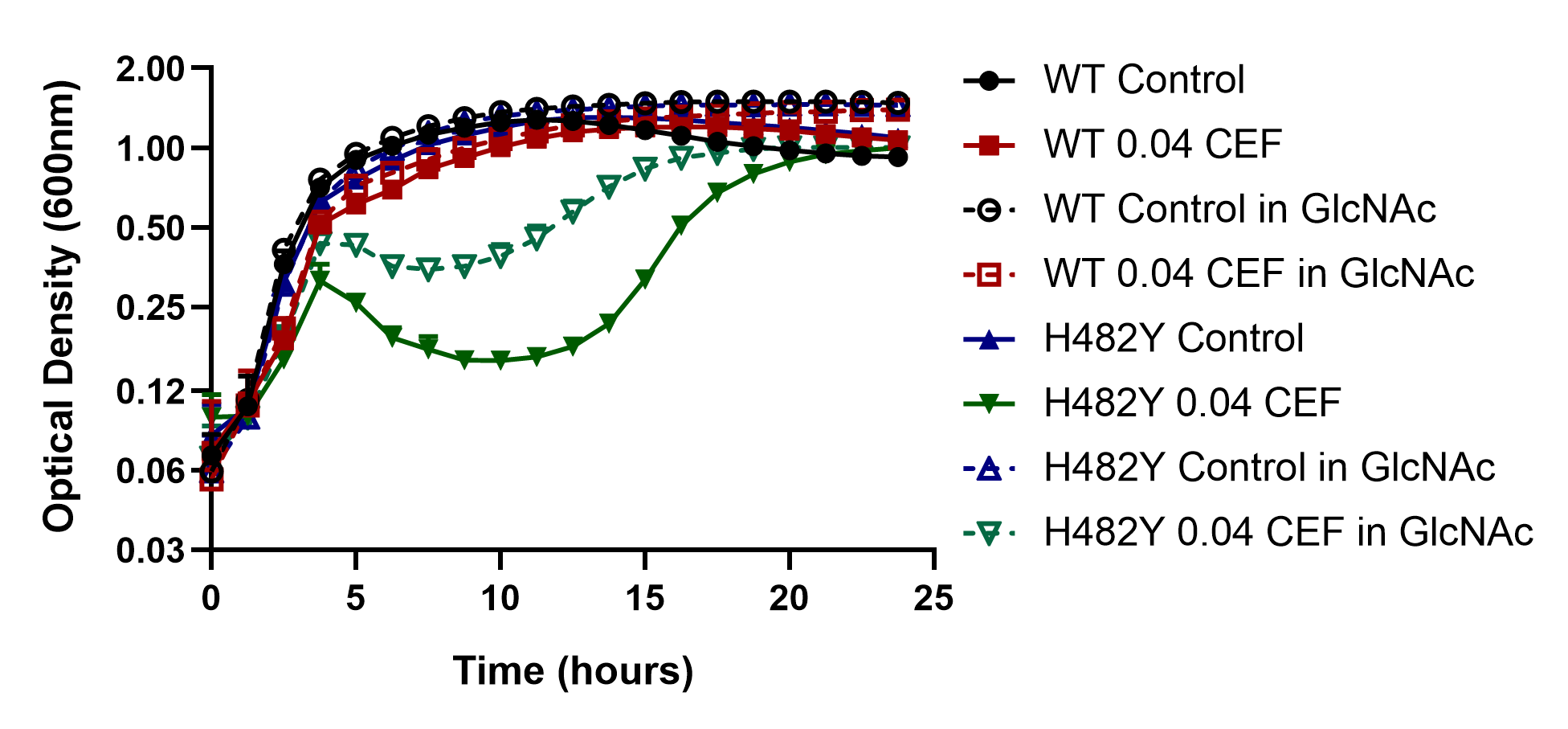

Supplement: FIG S4 [file mbio.03168-22-s0004.tif]

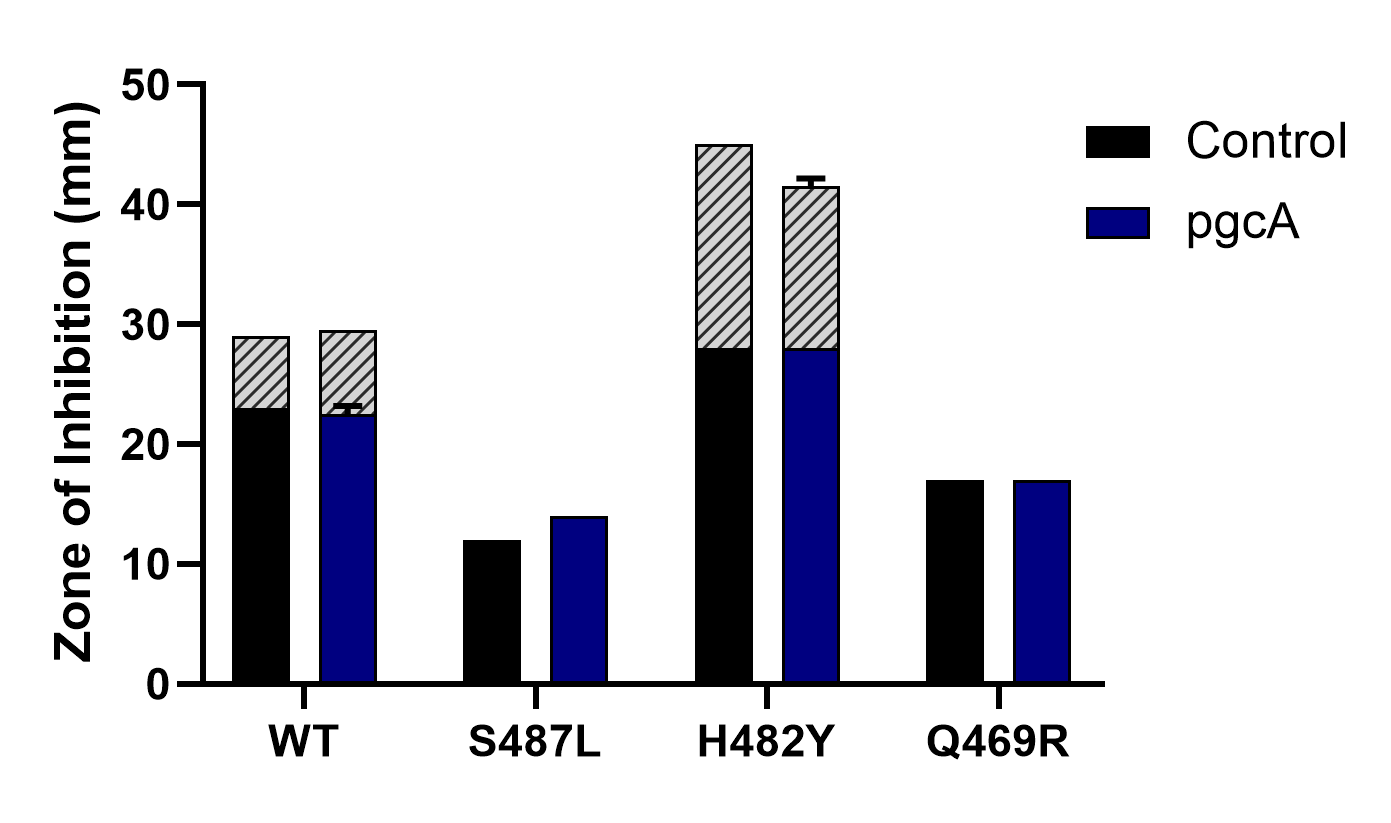

Supplement: FIG S5 [file mbio.03168-22-s0005.tif]
