# Supplementary material for: Mutations in rpoB That Confer Rifampicin Resistance Can Alter Levels of Peptidoglycan Precursors and Affect β-Lactam Susceptibility
Source: mBio. 2023 Feb 13;14(2):e03168-22. doi: 10.1128/mbio.03168-22 (PMC10128067; doi:10.1128/mbio.03168-22)
Supplement: TABLE S1 [file mbio.03168-22-s0006.docx]

**Supplementary Table 1** Bacterial strains used in the study

| **Strain number** | **Strain name** | **Additional details** |
| --- | --- | --- |
| HB26336 | Wild type *B. subtilis* 168 |  |
| HB26337 | 0.06R + 2.56C: 4th passage | Evolution; S487L (Strain A) |
| HB26338 | 0.12R + 2.56C: 4th passage | Evolution; S487L (Strain B) |
| HB26378 | 0.06R + 5.12C: 4th passage | Evolution; P520L (Strain C) |
| HB26266 | Q469R | Picked up from agar plates containing RIF |
| HB26341 | H482Y | Picked up from agar plates containing RIF |
| HB28012 | *ΔglmR* | *glmR::null* in HB26336 |
| HB28018 | S487L *ΔglmR* | *glmR::null* in HB26337 |
| HB28016 | H482Y *ΔglmR* | *glmR::null* in HB26341 |
| HB26492 | WT- P_spac(hy)_- glmM | lacA::P_spac(hy)_- glmM in WT using pPL82 |
| HB26498 | S487L- P_spac(hy)_- glmM | lacA::P_spac(hy)_- glmM in HB26337 using pPL82 |
| HB26496 | H482Y- P_spac(hy)_- glmM | lacA::P_spac(hy)_- glmM in HB26341 using pPL82 |
| HB26494 | Q469R- P_spac(hy)_- glmM | lacA::P_spac(hy)_- glmM in HB26266 using pPL82 |
| HB26482 | WT- P_spac(hy)_- pgcA* | lacA::P_spac(hy)_- pgcA(G47S) in WT using pPL82 |
| HB26488 | S487L- P_spac(hy)_- pgcA* | lacA::P_spac(hy)_- pgcA(G47S) in HB26337 using pPL82 |
| HB26486 | H482Y- P_spac(hy)_- pgcA* | lacA::P_spac(hy)_- pgcA(G47S) in HB26341 using pPL82 |
| HB26484 | Q469R- P_spac(hy)_- pgcA* | lacA::P_spac(hy)_- pgcA(G47S) in HB26266 using pPL82 |
| HB26472 | WT- P_spac(hy)_- pgcA | lacA::P_spac(hy)_- pgcA in WT using pPL82 |
| HB26478 | S487L- P_spac(hy)_- pgcA | lacA::P_spac(hy)_- pgcA in HB26337 using pPL82 |
| HB26476 | H482Y- P_spac(hy)_- pgcA | lacA::P_spac(hy)_- pgcA in HB26341 using pPL82 |
| HB26474 | Q469R- P_spac(hy)_- pgcA | lacA::P_spac(hy)_- pgcA in HB26266 using pPL82 |
| HB28124 | *ΔgamA* | *gamA::mls* in HB26336 |
| HB28127 | S487L *ΔgamA* | *gamA::mls* in HB26337 |
| HB28126 | H482Y *ΔgamA* | *gamA::mls* in HB26341 |
| HB28125 | *Q469R ΔgamA* | *gamA::mls* in HB26266 |
