# Supplementary material for: Mutations in rpoB That Confer Rifampicin Resistance Can Alter Levels of Peptidoglycan Precursors and Affect β-Lactam Susceptibility
Source: mBio. 2023 Feb 13;14(2):e03168-22. doi: 10.1128/mbio.03168-22 (PMC10128067; doi:10.1128/mbio.03168-22)
Supplement: TABLE S2 [file mbio.03168-22-s0007.docx]

**Supplementary Table 2** Primers used in the study

| **Primer Number** | **Primer Name** | **Primer Sequence** |
| --- | --- | --- |
| 9284 | rpoB-FP | GATGAAGTTTCCGTCGTTCAA |
| 9285 | rpoB-RP | GAAAATGCGTTCGCAGAATAG |
| 9286 | rpoB-seq-CI | CTTCTCCAGAACCAATTCCGT |
| 9728 | gamA-FP check | CAAGTCCGGCTACACCTTCT |
| 9729 | gamA-RP check | TGCAGAGCCAGTCTCACAGT |
| 9610 | glmS-qFP | AGAAAAAGGACGCATTGCAG |
| 9611 | glmS-qRP | TGAGCGTTCAGATAGCTTGG |
| 9614 | gamA-qFP | TCGGCTTGTATAAGCAGTTGA |
| 9615 | gamA-qRP | ACTTTGCGGATGAGATGGAG |
| 9616 | nagB-qFP | GCCTGCACCAAACTGAGAAT |
| 9617 | nagB-qRP | AAGTGATAGCTGTTCGGGTCA |
| 9612 | glmR-qFP | AATGTGCTTGCCGCTTTATC |
| 9613 | glmR-qRP | TCAAATTCCCGAGAGAATGG |
| 9608 | yvcJ-qFP | CCTTCATTGCTTCCGAAGTT |
| 9609 | yvcJ-qRP | TCAATCAGCCGGTCAAAAA |
| 9606 | glmM-qFP | CAAACAACGTCCAAAAGTGC |
| 9607 | glmM-qRP | ACTTCTGCGCCAATGGATAA |
| 9604 | glmU-qFP | GGTGCGGAAGAAGTGAAAAA |
| 9605 | glmU-qRP | AAGAAATGGCTGTGCCTGTT |
| 9618 | murA-qFP | CACAGACGCACGCATTTTAC |
| 9619 | murA-qRP | GGAGCAGATGTGCATTTTGA |
| 9620 | ponA-qFP | CGGAAAAGCGGCGGAAGAATT |
| 9621 | ponA-qRP | TTATCCGGGTTTTTGACCGGG |
| 8726 | gyrA-qFP | GGCGGCCATGCGTTATACAG |
| 8727 | gyrA-qRP | GCCATACCTACCGCAATGCC |
